# Discovery and Preclinical Development of Antigiardiasis Fumagillol Derivatives

**DOI:** 10.1128/AAC.00582-20

**Published:** 2020-09-21

**Authors:** Janak Padia, Liudmila Kulakova, Andrey Galkin, Osnat Herzberg

**Affiliations:** a Primetime Life Sciences, Germantown, Maryland, USA; b Institute for Bioscience and Biotechnology Research, University of Maryland, Rockville, Maryland, USA; c Department of Chemistry and Biochemistry, University of Maryland, College Park, Maryland, USA

**Keywords:** drug discovery, fumagillin, giardiasis

## Abstract

Giardiasis, caused by the intestinal parasite Giardia lamblia, is a severe diarrheal disease, endemic in poverty-stricken regions of the world, and also a common infection in developed countries. The available therapeutic options are associated with adverse effects, and G. lamblia resistance to the standard-of-care drugs is spreading. Fumagillin, an antimicrosporidiosis drug, is a therapeutic agent with potential for the treatment of giardiasis. However, it exhibits considerable, albeit reversible, toxicity when used to treat immunocompromised microsporidiosis patients. Fumagillin is also a highly unstable compound. To address these liabilities, we designed and synthesized stable fumagillol derivatives with lower levels of permeation across polarized epithelial Caco-2 cells and better potency against G. lamblia trophozoites than fumagillin. Metronidazole-resistant G. lamblia strains were also susceptible to the new fumagillol derivatives. In addition, these compounds were more potent against the amebiasis-causing parasite Entamoeba histolytica than fumagillin. Two compounds exhibited better thermal and acid stability than fumagillin, which should prolong the drug shelf life and reduce compound degradation in the stomach. Studies with a mouse model of giardiasis with the most stable compound, 4-(((((3*R*,4*S*,5*S*,6*R*)-5-methoxy-4-((2*R*,3*R*)-2-methyl-3-(3-methylbut-2-en-1-yl)oxiran-2-yl)-1-oxaspiro[2.5]octan-6-yl)oxy)carbonyl)amino)benzoic acid (compound 9), revealed that it had better efficacy (effective dose [ED]) than fumagillin at both the fully curative dose (the 100% ED) of 6.6 mg/kg of body weight and a 50% ED of 0.064 mg/kg. Plasma pharmacokinetics revealed the slow absorption of compound 9 through the gut, consistent with the *in vitro* characterization in Caco-2 cells. An acute-dose study yielded a maximum tolerated dose (MTD) of 1,500 mg/kg, 227-fold higher than the fully curative dose. Thus, along with improved stability, compound 9 also exhibited an excellent therapeutic window.

## Abstract (precis)

Giardiasis, caused by the intestinal parasite Giardia lamblia, is a severe diarrheal disease, endemic in poverty-stricken regions of the world, and also a common infection in developed countries. The available therapeutic options are associated with adverse effects, and G. lamblia resistance to the standard-of-care drugs is spreading. Fumagillin, an antimicrosporidiosis drug, is a therapeutic agent with potential for the treatment of giardiasis. However, it exhibits considerable, albeit reversible, toxicity when used to treat immunocompromised microsporidiosis patients.

## INTRODUCTION

Giardia lamblia is the causative agent of the diarrheal disease giardiasis, prevalent in poverty-stricken regions that lack adequate hygiene and safe water sources. G. lamblia is also the most common intestinal parasite in the United States and other developed countries, as it infects about 2% of the population (1–3). Chronic diarrhea leads to malnutrition, poses an economic burden on low-income families, and impairs the physical and cognitive development of children. The World Health Organization (WHO) included giardiasis in its Neglected Diseases Initiative in 2004 (4). Subsequently, in 2015, a WHO report on the global burden of foodborne diseases listed G. lamblia as a priority intestinal protozoan because of the high disease burden and the markedly increased frequency of citations from 1990 to 2008 (http://who.int/foodsafety/publications/foodborne_disease/fergreport/en/).

Metronidazole, tinidazole (a metronidazole analog), and nitazoxanide are the front-line drugs in the United States and developing countries used to treat giardiasis. These are generally effective, but they often produce undesirable side effects and, in some cases, require dose escalation or the resort to more toxic drugs, such as quinacrine (5). Moreover, G. lamblia strains refractive to the standard-of-care drugs have been observed in clinical isolates for a long time (6, 7), and the evolution of multiresistant strains has been demonstrated in the lab (6–9). The recurrence of giardiasis is widespread, with recurrence rates perhaps reaching 90% (10). For example, during a 2009 giardiasis outbreak in Norway, high-dose metronidazole treatment (up to 12 g) failed in 36% of cases, multiple rounds of metronidazole treatments were required, and 6% of still uncured patients needed hospitalization (11). A detailed giardiasis study in France reported metronidazole resistance as well as resistance to albendazole, a benzimidazole derivative standard-of-care drug used in Europe (12). These studies showed >20% multidrug resistance in both the patients and mice infected with the human drug-resistant G. lamblia strain. A need for alternative drugs is compounded by the fact that the prevention of infections through vaccines has been an unsolved challenge for many years.

The arsenal of approved drugs available for treating giardiasis belongs to only a few chemical classes, primarily the nitroimidazoles, thiazolides, and benzimidazoles. The nitroimidazoles are believed to be prodrugs activated by reduction of the nitro groups into free radicals, which cause cell damage (13); thiazolides are believed to act by inhibiting pyruvate:ferredoxin (flavodoxin) oxidoreductase (14); and the benzimidazoles inhibit the polymerization and assembly of microtubules (15, 16). Because G. lamblia drug resistance to these classes of compounds already exists, it is likely to show resistance to additional members of these classes. For example, resistance to a new 5-nitroimidazole, termed C17, with a side chain carrying a remote phenyl group in the 2 position of the imidazole ring, was induced in multiple G. lamblia strains (8). Thus, our strategy focuses on identifying new chemical scaffolds that target molecular processes different from those affected by current standard-of-care drugs and on minimizing side effects.

**FIG 1:**
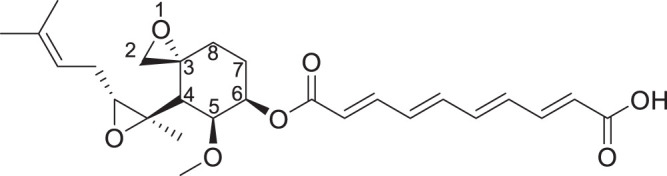
Fumagillin scheme showing the atom numbering convention.

To this end, we screened the NIH library of approved drugs for their ability to kill G. lamblia trophozoites and identified drugs that act by mechanisms different from those by which the currently approved antigiardiasis drugs act (17). Follow-up studies in a mouse giardiasis model revealed fumagillin to be one of the most promising drugs (18). Fumagillin (Fig. 1) was also an effective anti-intestinal amebiasis drug in a clinical setting, although it has not been approved for this indication (19). Fumagillin is a fermentation product of Aspergillus fumigatus. It effectively kills the trophozoites of common human G. lamblia strains, with a 50% inhibitory concentration (IC_50_) of 0.01 μM against the assemblage A WB strain, an IC_50_ of 0.002 μM against the assemblage B GS strain, and no apparent cytotoxicity toward mammalian CHO cells (IC_50_ > 100 μM) (17, 18). Proliferation assays measuring the minimum lethal concentration (MLC) in G. lamblia strains confirmed the potency of fumagillin and determined MLCs of 0.7 μM and 0.26 μM for the WB and GS strains, respectively (18).

Our mouse giardiasis model studies showed that the efficacy of fumagillin is superior to that of metronidazole (18). Moreover, 67 years ago, a U.S. Navy clinical study performed in Egypt found that orally administered fumagillin cured intestinal amebiasis and reported, somewhat anecdotally, that patients coinfected with G. lamblia were also cured (19). Fumagillin cures intestinal microsporidiosis caused by the protozoan Enterocytozoon bieneusi (20, 21) and has been approved by the European Union as an orphan antimicrosporidiosis drug for treating immunocompromised patients (22). It is also effective as a topical treatment of microsporidial keratoconjunctivitis (23); hence, it is recommended by the CDC for the treatment of refractive microsporidial eye infections. In contrast to the amebiasis patients, who exhibited minimal side effects during fumagillin treatment (19), 33% of the immunocompromised patients treated with fumagillin had a reversible side effect on bone marrow (primarily thrombocytopenia), with the spontaneous platelet count recovery occurring within 1 to 2 weeks after halting therapy.

Fumagillin drew renewed attention following the discovery that it inhibited angiogenesis in mice when administered systemically (24, 25). However, the inhibitory effect was accompanied by severe weight loss. The subsequent antiangiogenesis drug development effort has focused on reducing compound toxicity and increasing oral bioavailability (reviewed in reference 26). The observed weight loss inspired the development of fumagillin analogs for the treatment of obesity, which initially consisted of beloranib (27), which failed phase 3 clinical trials due to the death of two patients. A subsequent compound, ZGN-1061 (28), reached clinical trials, which were halted by the FDA because of cardiovascular concerns.

Fumagillin targets methionine aminopeptidase 2 (MetAP2) to form an irreversible covalent bond between the spiroepoxide group and an active-site histidine residue (29–32), whereas it is a weak inhibitor of methionine aminopeptidase 1 (MetAP1) (33). This activity might not be responsible for the inhibition of angiogenesis, as MetAP2 knockdown showed that fumagillin remained an effective endothelial cell growth inhibitor (34), and fumagillin also inhibited the expression of transcription factor Ets-1, a regulator of angiogenesis (35). Nevertheless, MetAP2 is expected to be essential to the survival of organisms that lack MetAP1 because the function of a number of proteins requires N-terminal methionine cleavage. MetAP1 and MetAP2 are both metallopeptidases that cleave the amino-terminal methionine, which is required for the activity of some proteins. The two MetAPs share the same overall fold and key catalytic residues (36, 37), yet they exhibit little amino acid sequence identity (38, 39). The human genome encodes both MetAP1 and MetAP2, whereas the G. lamblia genome encodes only MetAP2. MetAP2 from G. lamblia shares 38% amino acid sequence identity with the human enzyme, and the catalytic residues are invariant. Moreover, it has been shown that fumagillin inhibits MetAP2 from the parasites Plasmodium falciparum and Enterocytozoon bieneusi (29, 30), and the crystal structures of the fumagillin-MetAP2 complexes from human and E. bieneusi revealed the same mode of binding seen in human MetAP2 (29, 31). Because of the sequence homology and invariant catalytic machinery, it is plausible to assume that the G. lamblia MetAP2 is also inhibited by fumagillin, although this has not yet been demonstrated experimentally. Thus, we reason that the lack of MetAP1 in the G. lamblia genome implies that MetAP2 is likely essential to parasite survival and that enzyme inhibition by fumagillin would be lethal.

Fumagillin is an unstable compound, exhibiting light-, temperature-, humidity-, and pH-dependent degradation (40–43). Moreover, the C-6 ester bond (see Fig. 1 for the atom numbering) is amenable to hydrolysis by cellular esterases, including those produced by G. lamblia. While drugs can be easily protected from light using nontranslucent gel capsule formulations (44), the temperature sensitivity reduces the shelf life. Fumagillin requires refrigeration, a disadvantage that limits broad use in the clinic. Its instability at a pH comparable to that present in the stomach reduces the effective drug concentration by the time it reaches the intestine. This drug instability necessitates higher dosing, which in turn increases the potential for toxic effects. To enhance stability, we replaced the C-6 ester bond of fumagillin by stable bioisosteric groups. To reduce toxicity, we introduced C-6 side chains with various polar and nonpolar groups that can modulate absorption through the gut. The goal was to minimize gut absorption as much as possible and, thus, minimize drug-related toxicity while preserving transport into the trophozoites. Here, we report on the synthesis and evaluation of these fumagillol derivatives.

## RESULTS AND DISCUSSION

### Compound synthesis.

**FIG 2:**
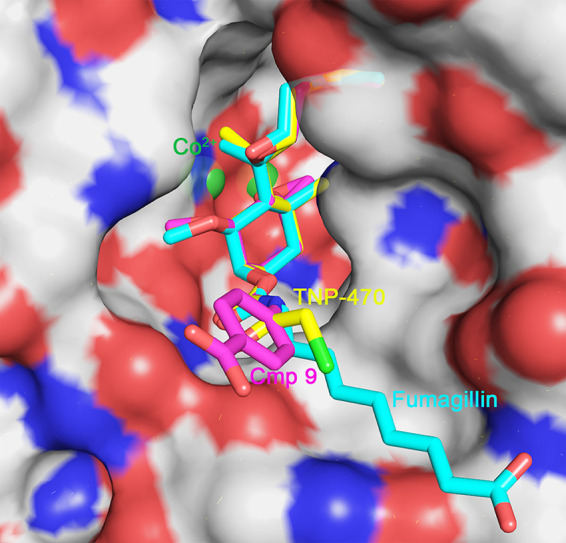
Binding of fumagillin, TNP-470, and compound 9 in the active site of MetAP2. The figure was generated with PyMOL software, using the coordinates of the proteins with PDB accession numbers 1BOA and 1B6A (31). The binding mode of compound 9 was generated by superpositioning the fumagillol core fragment on the respective core of TNP-470, including the carbamate moiety. The coloring scheme is as follows: red, oxygen; blue, nitrogen; bright green, chlorine; forest green, cobalt; cyan, fumagillin carbon; yellow, TNP-470 carbon; magenta, compound 9 carbon. The molecular surface around the active site is shown, with white, red, and blue corresponding to carbon, oxygen, and nitrogen atoms, respectively.

The current studies focus on the C-6 position of fumagillol because several crystal structures of MetAP2 bound with inhibitors show that the C-6 substituents are oriented toward the solvent. Hence, a wide range of groups can be accommodated (Fig. 2). Indeed, the development of antiangiogenesis fumagillin analogs with a modified C-6 tail shows that these modifications are tolerated and yield active compounds (45, 46). In contrast, the epoxide groups at C-3 and C-4 are buried deep in the active site, where the crowded environment offers limited replacement options. The ester group at the C-6 position is one of the most labile groups for compound stability. Fortunately, this position is the most amenable to synthetic modifications.

**FIG 3:**
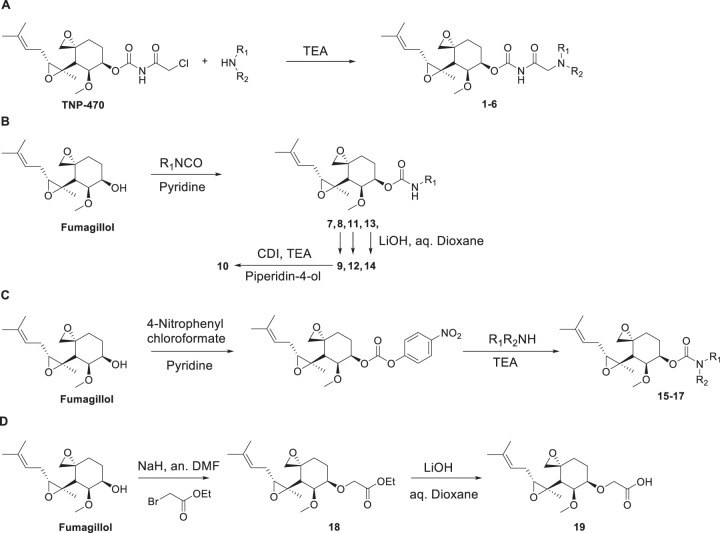
Synthetic routes of fumagillol derivatives. Compounds were prepared by one of the four synthetic approaches. (A) Compounds 1 to 6 were produced by alkylation of the appropriate amines with TNP-470 in the presence of trimethylamine. (B) Fumagillol was reacted with isocyanate reagents in the presence of pyridine to obtain the desired carbamate products (compounds 7, 8, 11, and 13), and hydrolysis of the ethyl ester derivatives (compounds 8, 11, and 13) with aqueous sodium hydroxide in dioxane yielded the corresponding carboxylic acid derivatives (compounds 9, 12, and 14, respectively). Coupling of compound 9 with piperidin-4-ol produced the corresponding amide derivative, compound 10. (C) Carbamate derivatives of secondary amines (compounds 15 to 17) were prepared by reacting the appropriate secondary amines with 4-nitrophenylcarbonate of fumagillol in the presence of pyridine. (D) Alkylation of fumagillol with 2-bromoacetate yielded compound 18, and subsequent hydrolysis of the ethyl ester produced the acid derivative, compound 19. TEA, triethylamine; *N*,*N*′-carbonyldiimidazole; an., anhydrous; aq., aqueous.

Figure 3 illustrates schematically the four synthetic routes used to prepare compounds 1 to 19. Table 1 provides the C-6 ester bioisosteric group (L) and the attached substituent group (R) of each compound and also includes the same information for the commercially available reference compounds fumagillol, fumagillin, and TNP-470. The general synthetic methods are provided in Materials and Methods, and detailed procedures for each compound are available in the supplemental material. Also provided in the supplemental material are the liquid chromatography (LC)-mass spectrometry (MS) and ^1^H nuclear magnetic resonance (NMR) data that validated the correct products.

**TABLE 1:**
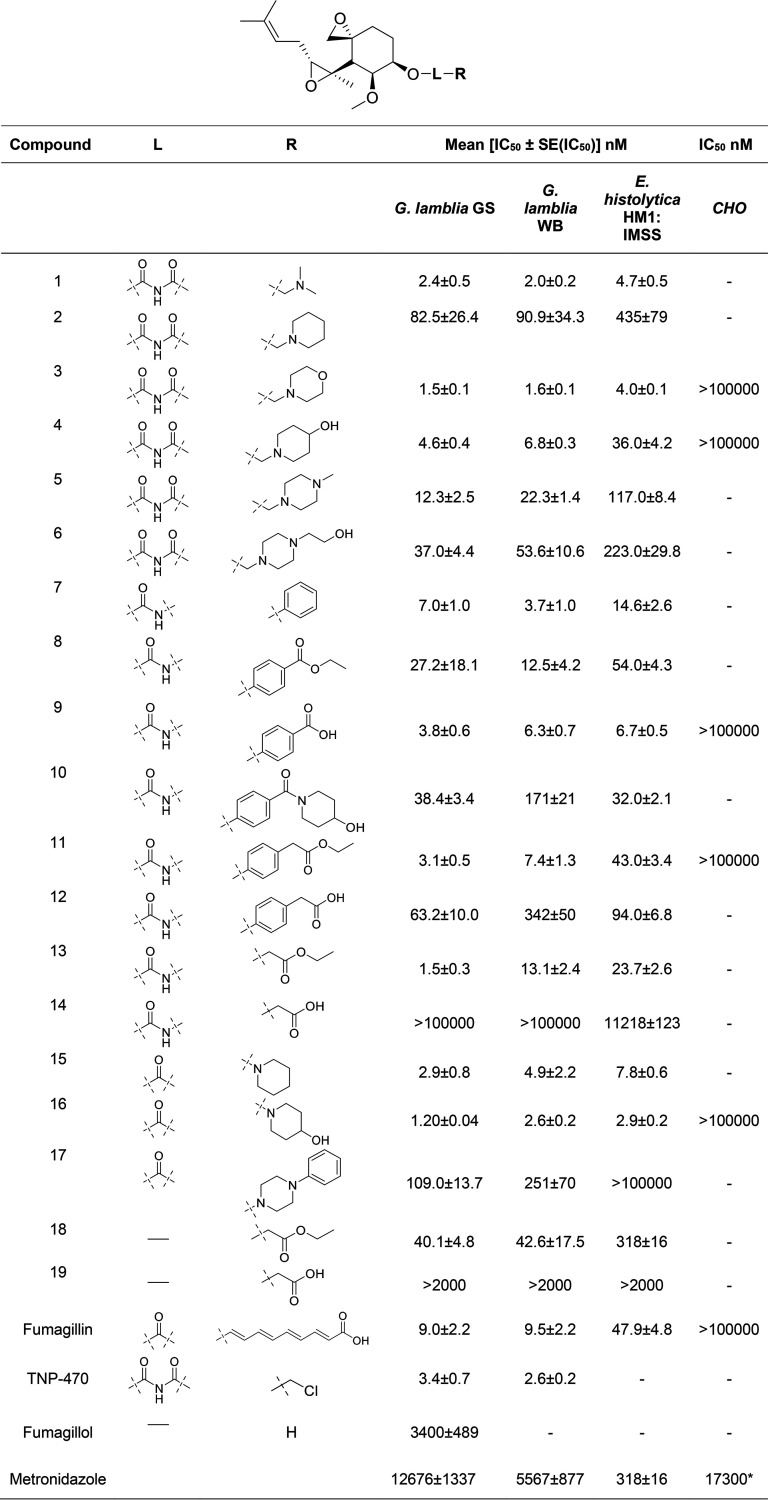
Synthesized and commercially available fumagillol derivatives and IC_50_ values^*a*^

a Values for metronidazole are included for comparison.*, the value is taken from reference 18.

Bioisosterism has been long used successfully in the design and development of drug candidates for improving potency, enhancing selectivity, altering compound physical properties and stability, reducing or redirecting metabolism, eliminating or modifying toxicophores, and acquiring novel intellectual property (47–54). The purpose of the bioisosteric replacement in the current study was to replace the fumagillin’s labile ester group with groups that are less sensitive to elevated temperature, humidity, pH, and hydrolytic enzyme activity. Indeed, Arico-Muendel et al. modified the C-6 ester group of fumagillin and reported compounds with improved pharmacokinetic profiles and activities against malaria parasites, trypanosomes, and amebas (55). In the current study, the bioisosteric groups replacing the ester group included acylcarbamate, carbamate, and ether. Concurrently, fumagillin’s 2,4,6,8-tetraenoic acid was replaced with groups with varied physicochemical properties to modulate the lipophilicity, charge, polarity, polar surface area, and number of rotatable bonds, as these properties are known to have an impact on transport across the epithelial cell barrier (56) and should also affect transport across the trophozoite cell membrane and compound potency in the parasite. As described below, the evaluations of the new compounds included the determination of *in vitro* potency, compound stability at elevated temperature and low pH, and permeation across Caco-2 cell membranes, as detailed below. The compound with the best *in vitro* characteristics and intellectual property potential was then advanced to *in vivo* studies.

### Compound potency: IC_50_ and MLC determination.

Cell viability assays are affected not just by the kinetics of the targeted protein (most likely MetAP2 in this case) but also by additional factors, such as compound permeation across the trophozoite membrane and the susceptibility of the compound to degrading cellular enzymes. These factors also affect compound efficacy *in vivo*. Additionally, both *in vitro* and *in vivo* cell viability may be affected by unknown off-target inhibition. Although the only validated target of fumagillol derivatives is MetAP2, the MetAP2-independent effects of fumagillin on angiogenesis are known (34, 35). As angiogenesis does not exist in G. lamblia, currently, there is no alternative MapAP2-independent activity of fumagillin that could be tested. Nevertheless, the possibility of a contribution of off-target inhibition to the lethal effects of drug candidates cannot be excluded.

Antigiardia assays followed by enzyme inhibition determination facilitated the selection of compounds for permeation and stability studies by *in vitro* methods, prior to advancing to *in vivo* studies. Compound potency was evaluated first by measuring G. lamblia trophozoite growth as a function of the compound concentration and calculating the IC_50_ values. Potency in Entamoeba histolytica trophozoites was also determined because of the early report that fumagillin cured amebiasis (19). The intent was to test whether the compounds would be suitable to treat gastrointestinal amebiasis rather than the invasive form of the disease, given that the designed strategy was based on reducing compound permeation across the epithelial cell barrier. As with the G. lamblia genome, the E. histolytica genome encodes only one MetAP, MetAP2, suggesting that the enzyme plays an essential role in the survival of the organism. Trophozoite growth was determined by monitoring the ATP cell content using a high-throughput homogeneous assay developed previously for G. lamblia (57). The ATP content serves as a marker of cell integrity since ATP degrades rapidly when cells die and the luminescence signal generated by luciferase requires ATP. The same assay was also adopted in 96-well plates for E. histolytica trophozoite growth measurement, as described in Materials and Methods. A similar E. histolytica trophozoite ATP content assay was described previously (58). Fumagillin, TNP-470, and a standard-of-care drug (metronidazole) served as positive controls, and cells without the drug (in the presence of 0.5% dimethyl sulfoxide [DMSO]) served as the negative control. Fumagillol exhibited low potency compared with the potencies of fumagillin, TNP-470, and many of the new compounds (Table 1). The potency of metronidazole was consistent with that described in many previous reports on the potency of G. lamblia (8, 17, 59–62) and ∼10-fold lower than that in the analogous E. histolytica ATP content assay reported previously (58). Thus, metronidazole exhibits much lower potency values than fumagillin and several of the newly synthesized fumagillol derivatives.

Despite the complexity of interpreting cell assays, the series of compounds provided insights into the structure-activity relationships (Table 1). First, all acylcarbamate derivatives (compounds 1 to 6) were active. The carbamate derivatives of primary amines (compounds 7 to 14) and secondary amines (compounds 15 to 17) were expected to be more stable than the acylcarbamate derivatives. Compound 7 with a phenyl ring exhibited excellent activity. The compound with a carboxylic acid ethyl ester substitution (compound 8) on the phenyl ring was ∼4-fold less active than compound 7, whereas the derivative with carboxylic acid (compound 9), a group with the potential to reduce permeation across Caco-2 cells, had potency comparable to that of compound 7 in both G. lamblia and E. histolytica. Compound 10, with an amide derivative, had decreased potency compared to that of compound 7. In order to optimize the location of the carboxylic group, we prepared compound 11 with a methylene group between the phenyl ring and the carboxylic ethyl ester group. It had potency comparable to that of compound 9 in G. lamblia trophozoites but potency lower than that of compound 9 in E. histolytica trophozoites. Interestingly, the use of a carboxylic acid group (compound 12) resulted in a loss of potency. The use of primary amines lacking the benzene on the R groups (compounds 13 and 14) showed that a carboxylic acid renders the compound inactive, perhaps because transport into the trophozoites was prevented by the increased polarity. Among the set of carbamate derivatives of cyclic secondary amine (compounds 15 to 17), compounds 15 and 16, with the simple cyclohexyl derivative and with the hydroxyl group substitution on the 4 position of the piperidine group, respectively, exhibited excellent potency. The more bulky and rigid R group of compound 17 led to a 100-fold loss of potency in G. lamblia trophozoites compared to the potency of compound 16 and rendered it inactive in E. histolytica trophozoites. The two compounds with an ether bioisosteric L group, compounds 18 and 19, had low potency, with the trend being similar to that for compounds 13 and 14; i.e., the compounds with the ester group were more active than those with the carboxylic acid.

Taken together, several of the newly synthesized compounds exhibited cell growth inhibition IC_50_ values comparable to or better than the IC_50_ of fumagillin in both the G. lamblia WB and GS strains. Similarly, several compounds were as potent as or more potent than fumagillin in E. histolytica. Compounds containing both acylcarbamate and carbamate bioisosteric linker groups exhibited excellent potencies, but those with the ether linker did not. Eight compounds (compounds 1, 3, 4, 7, 9, 13, 15, and 16) exhibited potencies superior to the potency of fumagillin in both pathogens, and in addition, compound 11 had potency superior to that of fumagillin in G. lamblia and comparable to that of fumagillin in E. histolytica (Table 1). We selected five compounds with two different bioisosteric groups, compounds 3 and 4 from the acylcarbamate class and compounds 9, 11, and 16 from the carbamate class, for further testing based on potency and hydrophobicity within the class.

The ATP cell content assay used to determine IC_50_ values does not differentiate between true lethality and metabolic arrest. The inhibition of metabolism enables the organism to resume proliferation once treatment is completed. To discriminate between the lethality of the promising compounds and the induction of metabolic inactivity by the promising compounds, the minimum lethal concentration (MLC) values were determined by treating the trophozoites with the test compounds for 3 days and transferring the resulting cultures to medium lacking the compounds for 3 days of proliferation. This assay was described previously for G. lamblia (18) and was adapted to E. histolytica in the current study. In addition to measuring MLC values in the G. lamblia WB and GS strains and in E. histolytica, the MLCs were also measured in metronidazole-resistant G. lamblia strains 713M3 and 1279-M1, to confirm that these compounds were also effective when the metronidazole resistance mechanism evolved. As can be seen in Table 2, fumagillin’s MLC values were 2 orders of magnitude higher than the IC_50_ values in all 4 G. lamblia strains (Table 1), consistent with the findings presented in our previous publication (18). Thus, even though fumagillin is a potent anti-G. lamblia drug, it might act in part by metabolic inhibition. Fumagillin’s IC_50_ and MLC values in E. histolytica were within the same range, supporting the true killing mechanism.

**TABLE 2:** MLC cell viability values

Compound	MLC (nM)				
	G. lamblia GS	G. lamblia WB	G. lamblia 713M3	G. lamblia 1279-M1	E. histolytica HM:IMSS
3	0.98	0.98	3.9	0.98	3.9
4	1.94	3.9	3.9	7.8	7.8
9	3.9	3.9	7.8	7.8	31.3
11	98	98			125
16	7.8	1.95	1.95	7.8	3.9
Fumagillin	250	125	250	125	78
TNP-470	3,100				
Fumagillol	2,500				
Metronidazole	12,500	7,700	100,000	100,000	1,500

In contrast to fumagillin, compounds 3, 4, 9, and 16 had comparable IC_50_ and MLC values in both G. lamblia and E. histolytica, consistent with true trophozoite killing and no metabolic inhibition component (Tables 1 and 2). Moreover, the MLC values confirmed that these four compounds were all effective against both metronidazole-responsive and -resistant G. lamblia strains, consistent with a mechanism of action that differs from that of metronidazole. However, compound 11 had substantially higher MLC values than IC_50_ values in G. lamblia, perhaps because it was degraded further by cellular proteases during the proliferation period to yield low-activity compound 12.

### CHO cell cytotoxicity.

Cytotoxicity in CHO-K1 (CHO) cells was also determined using the homogeneous ATP content assay with an ATPlite kit, to enable comparison with the IC_50_ values in the G. lamblia and E. histolytica trophozoites and provide an initial estimate of toxicity risk. Fumagillin served as a comparative control, untreated cells (including 0.5% DMSO) served as a negative control, and puromycin served as a positive control. The mean IC_50_ values of selected compounds for CHO cells are provided in Table 1. As can be seen, similar to fumagillin, all tested fumagillol derivatives had no significant cytotoxic effect on CHO cells. By comparison, metronidazole, a standard-of-care drug, exhibited considerable toxicity in CHO cells, as assessed by the poor selectivity vis-à-vis G. lamblia trophozoites (IC_50_ for CHO cells/IC_50_ for giardia < 10). The selectivity vis-à-vis E. histolytica was somewhat better (IC_50_ for CHO cells/IC_50_ for amebae > 10).

### *In vitro* MetAP2 inhibition.

As discussed above, the human genome encodes methionine MetAP2 and MetAP1, but the genomes of G. lamblia and E. histolytica encode only MetAP2. Fumagillin inhibits MetAP2, whereas it is a weak inhibitor of MetAP1. The crystal structures of MetAP2 from both human and the microsporidial parasite Enterocytozoon bieneusi contain binuclear zinc or cobalt catalytic centers (Fig. 2). Structures of the MetAP2 protein complexed with fumagillin or TNP-470 revealed covalently bound adducts through the spiroepoxide and a MetAP2 active-site histidine residue (29, 31). By analogy, the new fumagillol derivatives are expected to employ the same catalytic mechanism with MetAP2 from both G. lamblia and E. histolytica.

Despite much effort, we were unable to produce and purify soluble recombinant MetAP2 from G. lamblia and E. histolytica, using heterologous expression in Escherichia coli, baculovirus-Spodoptera frugiperda, and Leishmania tarentolae (LEXSY). To ensure that the new compounds act by the same mechanism employed by fumagillin, two representative compounds, one from the acylcarbamate series and the other from the carbamate series (compounds 3 and 9, respectively), were tested using commercially available purified human MetAP2. The IC_50_ values were comparable to those of fumagillin, consistent with the use of the same enzyme inhibition mechanism (Table 3). Based on the available crystal structures of MetAP2 complexes, the fumagillol core binds in a crowded environment, forming exquisite electrostatic interactions. Therefore, there is little room for the core fumagillol scaffold to adopt a different mode of binding. In contrast, the R group at C-6 can vary substantially because it is mostly solvent exposed. The L groups bind in a narrow site at the active-site entrance. The L groups of compounds 3 and 9 are bioisosteric to the fumagillin ester group, and their size and spatial differences could conceivably negatively affect enzyme inhibition properties. The MetAP2 inhibition data showed only a small effect, as all 3 compounds had IC_50_ values within the nanomolar range. A model of compound 9 bound by analogy to the experimentally determined binding modes of fumagillin and TNP-470 supports the hypothesis that the MetAP2 active site can accommodate the ester bioisosteric group (Fig. 2).

**TABLE 3:** Inhibition of human MetAP2

Compound	Mean ± SE IC_50_ (nM)	IC_50_ of compound/IC_50_ of fumagillin
3	0.67 ± 0.02	1.68
9	1.10 ± 0.30	2.75
Fumagillin	0.40 ± 0.03	1

Taken together, both compounds 3 and 9 exhibit trophozoite killing properties superior to those of fumagillin, while they are also excellent inhibitors of MetAP2. Factors that contribute to the ∼100-fold improved MLCs compared with the MLC of fumagillin may include better uptake into the trophozoites and stabilization due to the ester bioisosteres at the C-6 position that are resistant to hydrolysis by trophozoite esterases. Multiple reasons other than enzyme inactivation may contribute to the large differences in potency of the compounds between giardia trophozoites and CHO cells, including differences in the membrane structures of these cells; the presence of an alternative MetAP, MetAP1, in human cells but not in G. lamblia; and differences between degrading enzymes in human and giardia.

### Caco-2 cell monolayer permeation.

Next, we tested the permeation of the compounds across polarized Caco-2 cell monolayers, a method that is widely used as an *in vitro* model of the human small intestinal mucosa to predict the absorption of orally administered drugs (63, 64). With noninvasive intestinal infections, there is no need to establish a high plasma concentration. Since a high rate of uptake through the host gut lining could increase compound toxicity, we sought to reduce gut absorption. Fumagillin and two potent compounds, each with a different L group (compounds 3 and 9), were analyzed in Caco-2 epithelial cell (ATCC) monolayers together with two standard compounds, propranolol (a permeant control) and atenolol (a nonpermeant control). Both apical-to-basal and basal-to-apical permeation values were measured to obtain information about efflux. The apparent permeation coefficients are summarized in Table 4. Both compounds 3 and 9 had lower Caco-2 cell permeation values than fumagillin (permeation, 62% and 53% of the permeation of fumagillin, respectively). For all three compounds, the efflux ratio was ∼2, indicating that, in contrast to the control compounds, which had efflux ratios of <0.5, the fumagillol derivatives, including fumagillin, may be subject to active efflux. Expelling drugs into the intestinal tract should reduce the systemic toxic effects and increase the effective drug concentration at the site of parasite infection, which is highly desirable. The efflux machinery is currently unknown.

**TABLE 4:** Caco-2 cell monolayer permeation data

Compound	Mean ± SE *P*_app_ (10^−6^ cm/s)		Efflux ratio
	Apical to basal	Basal to apical	
3	15.78 ± 0.94	30.40 ± 4.35	1.95
9	12.63 ± 0.63	23.58 ± 8.14	1.90
Fumagillin	23.67 ± 2.52	48.82 ± 9.13	2.13
Propranolol	22.57 ± 1.10	8.14 ± 0.28	0.36
Atenolol	0.32 ± 0.00	0.14 ± 0.14	0.45

### Compound stability.

**FIG 4:**
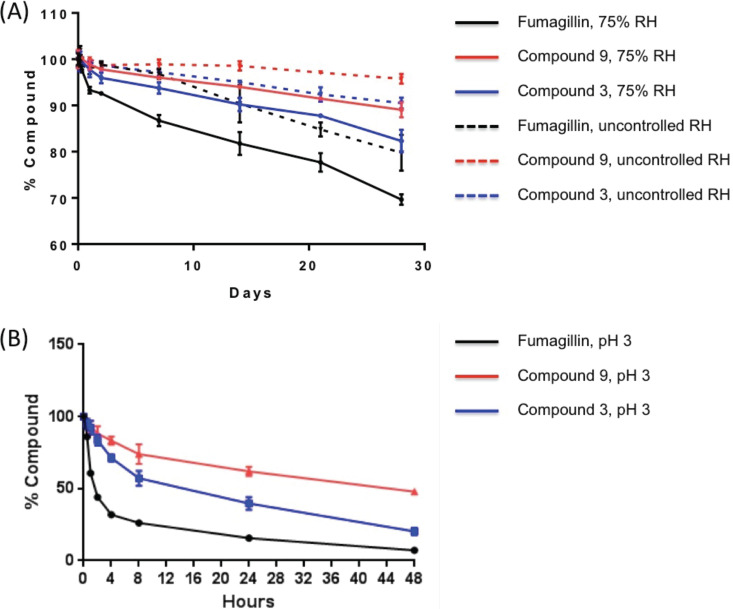
Compound degradation at 37°C and 75% or uncontrolled relative humidity (RH) (A) and at pH 3 and 25°C (B). The bars depict the mean errors.

Accelerated temperature tests were performed at 37°C and under 75% relative humidity or laboratory uncontrolled relative humidity, comparing compounds 3 and 9 to fumagillin. Sanofi reported previously that fumagillin degraded from 98% to 85% purity when stored at 40°C for 2 weeks at ambient humidity (42). The experiments reported here showed that at 37°C and at both 75% and ambient humidity, fumagillin degraded substantially more rapidly than compounds 3 and 9 (Fig. 4). At day 28, 20.22% and 30.37% of the fumagillin sample was degraded at uncontrolled and 75% relative humidity, respectively, whereas compound 9 was degraded by 4.22% and 10.95%, respectively, and compound 3 was degraded by 9.50% and 17.67%, respectively. The enhanced temperature stability predicts a substantially improved shelf life of compounds 3 and 9 compared with that of fumagillin.

Similarly, both compounds 3 and 9 were more stable than fumagillin at pH 3, which mimics the pH in the stomach (Fig. 4). The rapid degradation at pH 3 allowed half-life determinations, which were 1.42, 17.25, and 43.21 h, for fumagillin, compound 3, and compound 9, respectively (Table 5). The dramatic improvement in stability is not surprising, because the ester group of fumagillin is susceptible to hydrolysis at low pH. Thus, orally delivered compounds 3 and 9 should be more stable than fumagillin. This should enable the administration of lower doses, reducing toxicity risks.

**TABLE 5:** Compound stability under specified environmental conditions

Compound	Mean ± SE degradation (%) at 4 wk and 37°C at:		Mean ± SE half-life (h) at pH 3 and 25°C
	Uncontrolled humidity	75% humidity	
3	9.50 ± 1.18	17.67 ± 2.42	17.25 ± 2.26
9	4.22 ± 1.04	10.95 ± 1.66	43.21 ± 1.45
Fumagillin	20.22 ± 3.90	30.37 ± 1.12	1.42 ± 0.05

### Compound efficacy in mouse giardiasis model.

**FIG 5:**
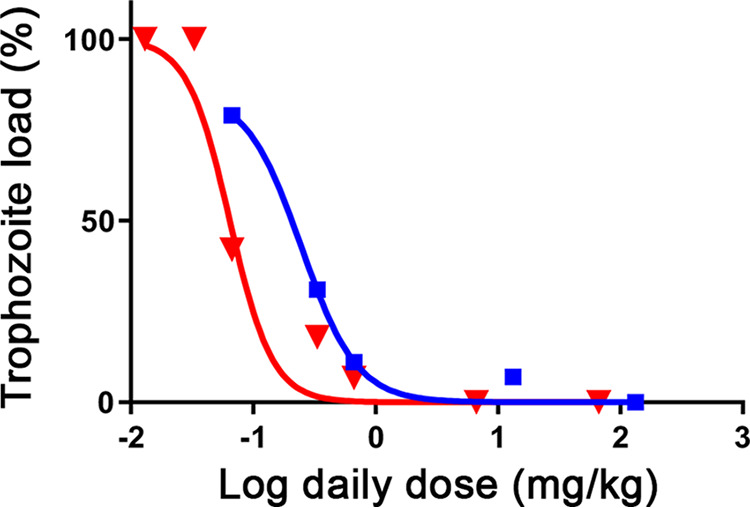
Efficacies of compound 9 (red) and fumagillin (blue) in the mouse giardiasis model. The dose-responses (triangles and squares) were fitted to sigmoidal curves using the computer program GraphPad Prism (version 8.2.1; GraphPad Software Inc., La Jolla, CA). The EC_50_ values for a 4-day single-daily-dose treatment regimen were 0.064 and 0.247 mg/kg/day for compound 9 and fumagillin, respectively.

We previously used a mature C57BL/6J mouse giardiasis model to show that fumagillin was by far more efficacious than the standard-of-care drug metronidazole (18). We selected compound 9 for efficacy studies because of its superior stability not just compared with that of fumagillin but also compared with that of compound 3. The C57BL/6J mice were infected with G. lamblia GS trophozoites, treated for 4 days, and euthanized as described in Materials and Methods. Surviving trophozoites were allowed to proliferate *in vitro* for 6 days in order to determine the dose required for a complete cure (the 100% effective dose [ED_100_]) and to obtain ED_50_ values (Fig. 5). Five doses of fumagillin were sufficient to fit a trophozoite load curve and calculate the ED_50_. For compound 9, seven doses were necessary to span the remaining trophozoite loads higher and lower than 50% and to calculate the ED_50_. These studies showed that daily administration of compound 9 at 6.6 mg/kg of body weight cured all 10 treated mice, as trophozoites did not proliferate in the subsequent *in vitro* assays. In contrast, in the case of fumagillin, none of the mice were cured and trophozoites survived and proliferated at 2 times a dose of 13.7 mg/kg, with 7% of the trophozoite load remaining compared with that in the untreated control mice. The low dose of compound 9 that completely cleared the infection is an important efficacy indicator because all G. lamblia trophozoites must be eliminated to prevent the recurrence of the disease. The better efficacy of compound 9 than of fumagillin was also manifested in the 4-fold lower ED_50_ values for compound 9 than for fumagillin, calculated to be 0.064 and 0.247 mg/kg, respectively, and in *R*^2^ values of 0.9507 and 0.9882 for compound 9 and fumagillin, respectively.

### Plasma pharmacokinetics.

**FIG 6:**
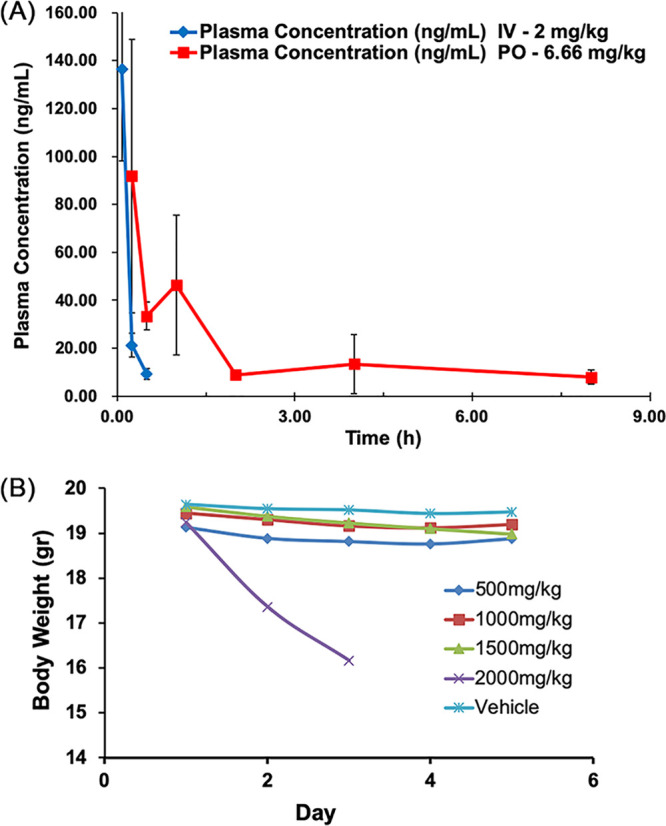
Mouse pharmacokinetic and toxicity studies. (A) Time course of the compound 9 plasma concentration in female C57BL/6 mice following oral and intravenous administration at 2 mg/kg and 6.6 mg/kg, respectively. Data represent the means ± SD of three replicates at each point. (B) Body weight changes in female C57BL/6 mice after oral administration of a single dose of compound 9. Data points represent averages for six animals. The experiment with the highest dose (2,000 mg/kg) was terminated on day 3, following the unexpected death of two animals. The remaining animals were sacrificed, and the organs from three mice were processed for histopathology examination.

The mean plasma concentration-time profiles for intravenous (i.v.) and *per os* (p.o.) administration in C57BL/6 female mice are shown in Fig. 6A. Following administration of a dosage of compound 9 at 2 mg/kg via an i.v. bolus, plasma concentrations reached ∼136 ng/ml at the 5-min time point and then decreased rapidly to below the limit of quantification (<5 ng/ml) by 1 h after dosing. Based on these data, the calculated half-life was 7.2 min. In contrast, at the earliest time point (15 min) following oral administration of a 6.6-mg/kg bolus, the plasma concentration reached a maximum of ∼92 ng/ml and then decreased gradually and remained detectable for 8 h. These data suggest a slow absorption across the intestinal cell wall, a desirable property for a drug targeting intestinal pathogens.

### MTD determination.

**FIG 7:**
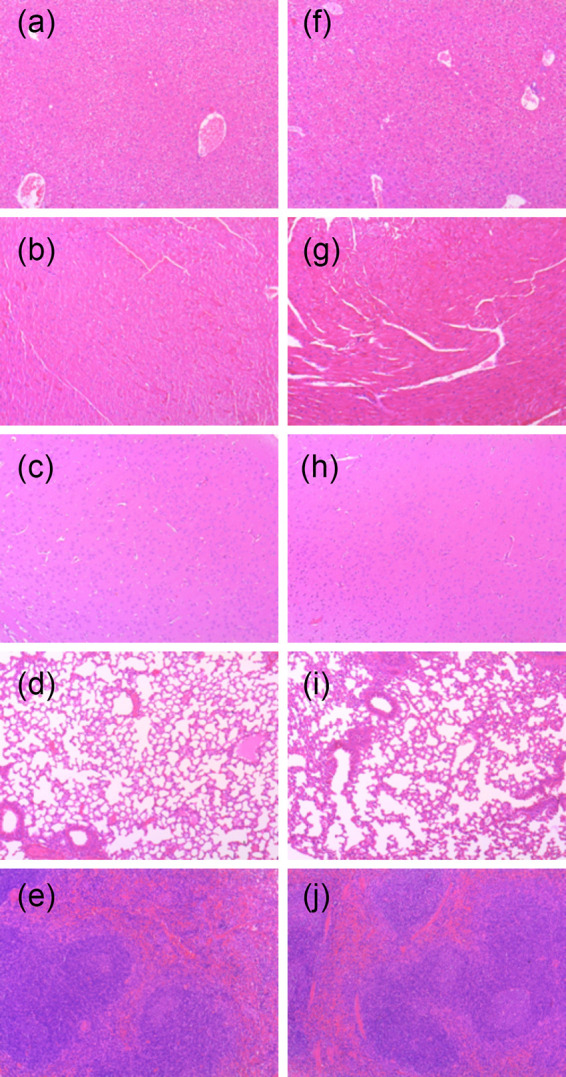
Histological appearance of representative hematoxylin-eosin-stained mouse tissues treated with 2,000-mg/kg compound 9 (a to e) or with vehicle alone (f to j). (a and f) Liver; (b and g) heart; (c and h) brain; (d and i) lung; (e and j) spleen.

Single-dose acute toxicity studies with C57BL/6 female mice (replicates of six) revealed no weight change and no negative clinical signs for oral administration at doses of up to 1,500 mg/kg. The group administered 2,000-mg/kg compound showed an ∼15% reduced weight on day 3 (Fig. 6B), and two mice died unexpectedly. Consequently, the study was halted, indicating that the 50% lethal dose (LD_50_) was >2,000 mg/kg. The tissues of three surviving mice were analyzed along with the tissues of three animals treated with the vehicle alone. The histopathology examination of hematoxylin-eosin-stained tissues from five major organs (liver, heart, brain, lung, and spleen) under a light microscope at a 100-fold magnification revealed normal tissue with no significant changes compared with the control tissues (Fig. 7). Based on these findings, the maximum tolerated dose (MTD) value was determined to be 1,500 mg/kg. The curative dose that killed all trophozoites was 6.6 mg/kg, 227-fold lower than the MTD value, indicating an excellent therapeutic window. Assuming that the mouse LD_50_ is >2,000 mg/kg, the therapeutic index of compound 9 is LD_50_/ED_50_ = 2,000/0.064 > 31,250, also indicative of an excellent safety profile.

### Conclusion.

Resistance to frontline antigiardiasis drugs is spreading, and these therapeutics often fail because of patient noncompliance due to undesirable side effects. To offer alternative therapeutic options, we have begun the development of drug candidates derived from the fumagillol scaffold. These inhibit G. lamblia MetAP2, the sole MetAP produced by the organism. MetAP2 has not been targeted by current standard-of-care antigiardiasis drugs; therefore, inhibitors of the enzyme should circumvent existing drug resistance mechanisms. The fumagillol derivative 4-(((((3*R*,4*S*,5*S*,6*R*)-5-methoxy-4-((2*R*,3*R*)-2-methyl-3-(3-methylbut-2-en-1-yl)oxiran-2-yl)-1-oxaspiro[2.5]octan-6-yl)oxy)carbonyl)amino)benzoic acid (compound 9) exhibited better stability at elevated temperatures and at low pH than the orphan drug, fumagillin, used to treat microsporidiosis in immunocompromised patients. Based on its properties, compound 9 is expected to have a better shelf life and a lower susceptibility to degradation in the stomach than fumagillin. Its lower permeation across the Caco-2 epithelial cell barrier model, together with its higher potency in trophozoites, adds to the advantages of compound 9. These properties were recapitulated in the *in vivo* studies reported here. Mouse giardiasis model studies showed the better efficacy of compound 9 than of fumagillin, and early pharmacokinetic/toxicity studies in mice showed slow gut absorption and a wide therapeutic window. We conclude that compound 9 shows promise that makes it worthy of further development as an antigiardiasis agent. Potentially, the new antigiardiasis drug may also be used in combination with existing drugs to reduce the probability of emergence of a resistance mechanism.

## MATERIALS AND METHODS

### Chemistry.

All materials were obtained from commercial suppliers and used without further purification. All solvents were dried using an aluminum oxide column. Thin-layer chromatography was performed on precoated silica gel 60 F254 plates. Purification of intermediates was carried out by normal-phase column chromatography. NMR (400-MHz) spectra were recorded using a Bruker Advance spectrophotometer. LC-MS analysis was performed using a Waters Acquity ultraperformance liquid chromatography system equipped with a photodiode array detector, an evaporative light-scattering detector (ELSD), and a Micromass LCT Premier electrospray ionization time-of-flight mass spectrometer. The mobile phases used for these assays were 0.1% formic acid in water (mobile phase A) and 0.1% formic acid in acetonitrile (mobile phase B). A BEH C_18_ column (particle size, 1.7 μm; 2.1 mm by 100 mm; Waters) was used to analyze the samples at a linear gradient of from 90% mobile phase B to 10% mobile phase B over 5 min at a flow rate of 0.4 ml/min. ELSD parameters were set as follows: gain, 500; column heater temperature, 35°C; pressure, 50 lb/in^2^; drift tube temperature, 50°C; nebulizer, heating (50%). All derivatives tested for biological activity showed >95% purity by high-performance liquid chromatography (HPLC) analysis (with detection using ELSD).

### Compound synthesis.

Fumagillol was purchased from JR MediChem LLC, and TNP-470 was purchased from Sinova Inc. Detailed synthetic procedures for each compound along with compound validation are provided in the supplemental material.

### (i) Synthesis of 6-*O*-dialkylaminoacetylcarbamoylfumagillol derivatives (compounds 1 to 6) (Fig. 3A, general method A).

To a stirred solution of (3*R*,4*S*,5*S*,6*R*)-5-methoxy-4-((2*R*,3*R*)-2-methyl-3-(3-methylbut-2-en-1-yl)oxiran-2-yl)-1-oxaspiro[2.5]octan-6-yl (2-chloroacetyl)carbamate (TNP-470) (1 eq) and triethylamine (1 or 2 eq) in toluene was added amine (1 eq), and the mixture was stirred at room temperature for 18 h. The solvents were evaporated under reduced pressure, reconstituted with ethyl acetate, washed with water and brine, and dried with anhydrous sodium sulfate. The solvent was removed under reduced pressure, and the residue was purified by silica gel column chromatography (the eluent was 2.5% methanol in ethyl acetate) to give the desired product.

### (ii) Synthesis of 6-*O*-(*N*-substituted carbamoyl) fumagillol derivatives (compounds 7, 8, 11, and 13) (Fig. 3B, general method B).

To an ice-cold stirred solution of fumagillol (1 eq) in dichloromethane was added dropwise isocyanate (1.5 eq), followed by addition of pyridine (2 eq). The mixture was stirred at 10 to 15°C for 2 h and then at room temperature for 15 h. The reaction mixture was concentrated under reduced pressure. The residue was dissolved in ethyl acetate, washed with water and saturated aqueous sodium chloride solution, and dried over anhydrous magnesium sulfate. The solvent was removed under reduced pressure, and the residue was subjected to silica gel column chromatography, using ethyl acetate and hexane.

### (iii) Synthesis of 6-*O*-(*N*-substituted carbamoyl) fumagillol derivatives (compounds 15 to 17) (Fig. 3C, general method C).

To an ice-cold stirred solution of fumagillol (1 eq) and pyridine (1.5 eq) in dichloromethane was slowly added 4-nitrophenyl chloroformate (1.2 eq). The mixture was stirred at 10 to 15°C for 1 h and then at room temperature for 4 h. The reaction mixture was diluted with dichloromethane, washed with water and saturated aqueous sodium chloride solution, and dried over anhydrous magnesium sulfate. The solvent was removed under reduced pressure, and the residue was subjected to silica gel column chromatography, using ethyl acetate and hexane, to get the intermediate (3*R*,4*S*,5*S*,6*R*)-5-methoxy-4-((2*R*,3*R*)-2-methyl-3-(3-methylbut-2-en-1-yl)oxiran-2-yl)-1-oxaspiro[2.5]octan-6-yl (4-nitrophenyl)carbamate as a yellow oil (63%).

To a stirred reaction mixture of (3*R*,4*S*,5*S*,6*R*)-5-methoxy-4-((2*R*,3*R*)-2-methyl-3-(3-methylbut-2-en-1-yl)oxiran-2-yl)-1-oxaspiro[2.5]octan-6-yl (4-nitrophenyl)carbamate (1 eq) and trimethylamine (4 eq) in dichloromethane was added secondary amine (4 eq). The mixture was stirred for 15 h at room temperature. The reaction mixture was concentrated under reduced pressure. The residue was dissolved in ethyl acetate, washed with water and saturated aqueous sodium chloride solution, and dried over anhydrous magnesium sulfate. The solvent was removed under reduced pressure, and the residue was subjected to silica gel column chromatography, using a gradient solvent if 1 to 2.5% methanol in ethyl acetate, to get the desire carbamate product.

### (iv) Synthesis of ethyl 2-(((3*R*,4*S*,5*S*,6*R*)-5-methoxy-4-((2*R*,3*R*)-2-methyl-3-(3-methylbut-2-en-1-yl)oxiran-2-yl)-1-oxaspiro[2.5]octan-6-yl)oxy)acetate (compound 18) (Fig. 3D, general method D).

To a stirred ice-cold solution of fumagillol (226 mg, 0.8 mmol) in anhydrous dimethylformamide (DMF) (5 ml) was added 60% sodium hydride (46 mg, 1.2 mmol), followed by the slow addition of ethyl 2-bromoacetate (200 mg, 1.2 mmol) in 0.5 ml of anhydrous DMF. The mixture was stirred at 10 to 15°C for 1 h and then allowed to stir at room temperature for 16 h. The reaction mixture was concentrated under reduced pressure. The residue was dissolved in ethyl acetate, washed with water and saturated aqueous sodium chloride solution, and dried over anhydrous magnesium sulfate. The solvent was removed under reduced pressure, and the residue was subjected to silica gel column chromatography (the eluent was hexane-ethyl acetate [4:1]) to give 146 mg the desired compound as an oil (49% yield).

### (v) General method for synthesis of carboxylic acid derivatives (compounds 9, 12, 14, and 19).

To a stirred solution of ethyl ester derivative (1 eq) in dioxane was added lithium hydroxide (4 eq) in 0.2 ml water. The reaction mixture was stirred for 14 h, and the pH was adjusted to 5 with dilute HCl. The reaction mixture was concentrated under reduced pressure, and the residue was subjected to silica gel column chromatography, using ethyl acetate and hexane, to get the corresponding acid.

### (vi) Synthesis of (3*R*,4*S*,5*S*,6*R*)-5-methoxy-4-((2*R*,3*R*)-2-methyl-3-(3-methylbut-2-en-1-yl)oxiran-2-yl)-1-oxaspiro[2.5]octan-6-yl (4-(4-hydroxypiperidine-1-carbonyl)phenyl)carbamate (compound 10).

To a stirred solution of 4-(((((3*R*,4*S*,5*S*,6*R*)-5-methoxy-4-((2*R*,3*R*)-2-methyl-3-(3-methylbut-2-en-1-yl)oxiran-2-yl)-1-oxaspiro[2.5]octan-6-yl)oxy)carbonyl)amino)benzoic acid (compound 9) (49 mg, 0.11 mmol) and triethylamine (25 mg, 0.25 mmol) in dichloromethane (DCM; 5 ml) was added carbonyldiimidazole (27 mg, 0.17 mmol). The resulting reaction mixture was stirred at room temperature for 1 h under nitrogen and then treated with a solution of piperidin-4-ol (22 mg, 0.22 mmol) in DCM (1 ml), and the stirring was continued at room temperature for 14 h. The reaction mixture was concentrated under reduced pressure. The residue was dissolved in ethyl acetate (20 ml), washed with water and saturated aqueous sodium chloride solution, and dried over anhydrous magnesium sulfate. The solvent was removed under reduced pressure, and the residue was subjected to silica gel column chromatography (the eluent was hexane-ethyl acetate [1:1]) to give 21 mg of a yellow solid (36% yield).

### Trophozoite cultures. (i) Giardia cultures.

Trophozoites of G. lamblia isolates WB and GS were grown anaerobically in borosilicate glass screw-cap culture tubes (Fisher Scientific) at pH 7.0 in Keister’s modified TYI-S-33 medium (ATCC). The medium was supplemented with 10% heat-inactivated bovine serum (Sigma-Aldrich) and 0.05% bovine bile (Sigma-Aldrich). To attain low-oxygen-tension conditions, the tubes were filled to 85% to 90% of their total volume capacity and incubated without shaking at 37°C. Subcultures (2 × 10^5^ trophozoites per tube) were made three times a week. Detachment of trophozoites for inoculation was achieved by chilling the cultures on ice for 20 min. Culturing and detachment of metronidazole-resistant G. lamblia assemblage A 713M3 and assemblage B 1279-M1 trophozoites followed the same protocol, except that the growth medium was supplemented with metronidazole, with the concentration gradually being increased to 10 μM.

### (ii) Ameba cultures.

E. histolytica strain HM1:IMSS trophozoites were grown at 37°C in TYI-S-33 medium supplemented with penicillin (100 U/ml) and streptomycin sulfate (100 μg/ml) (65). Trophozoites were grown anaerobically in borosilicate tubes or T25 flasks, and subcultures were made 1 to 2 times a week. Trophozoites were detached for inoculation by chilling the cultures on ice for 20 min.

### Trophozoite growth inhibition IC_50_ determination.

The growth inhibition assays were performed in duplicate, once for each compound, for all compounds except fumagillin, which served as a positive control and whose inhibitory ability was measured multiple times as new sets of compounds were tested. All dry compounds except for fumagillin were dissolved in dimethyl sulfoxide (DMSO; Sigma) at a stock concentration of 10 mM and then diluted 1:100 in growth medium to a final compound concentration of 100 μM; fumagillin was diluted to 20 μM. Compound solution aliquots (100 μl) were prepared by 3-fold serial dilutions in growth medium containing 0.5% DMSO and distributed into 96-well assay plates. This was followed by the addition of 10 μl G. lamblia or E. histolytica culture containing 10,000 trophozoites. Fumagillin served as a positive control, and the medium-diluted DMSO alone served as a negative control. The assay plates were placed in a BD GasPak EZ container system (BD Diagnostics) to create an anaerobic growth environment. The sealed containers were incubated at 37°C for 72 h. Following incubation, 70 μl/well of the ATPlite reagent (PerkinElmer) was added to the assay plates for one-step lysis and ATP level detection. The luminescent signals of the assay plates were measured on an EnSpire 2300 plate reader (PerkinElmer). Concentration-response titration points for each compound were fitted to a 4-parameter logistic nonlinear regression model using the KaleidaGraph tool, yielding the IC_50_ value and standard error (SE).

### Trophozoite viability MLC determination. (i) Giardia assay.

Compounds were prepared by 2-fold serial dilutions in growth medium containing 0.5% DMSO (diluted from a starting concentration of 1 μM to 12 concentrations for all compounds except metronidazole, fumagillol, and TNP-470, which were diluted from 100 μM), and 100 μl/well was transferred in duplicate to the 96-well culture plate. Ten microliters of G. lamblia trophozoites was plated at a density of 10,000 cells/well. The plates were incubated under anaerobic condition in a BD GasPak EZ container system (BD Diagnostics) at 37°C for 72 h and surveyed visually under a microscope to check trophozoite survival, mobility, and attachment. In all cases, duplicate measurements yielded identical growth/death transitions. The plates were chilled on ice for 30 min, and the entire content of each of 4 wells within the growth/death transition of one of the duplicate experiments was transferred for proliferation into the 8-ml tubes containing growth medium and no drug. The tubes were incubated under anaerobic condition for 3 days at 37°C and checked under a microscope. The MLC value was the concentration in the tube with the lowest compound concentration without any live organism.

### (ii) Ameba assay.

For the MLC assay, compounds were prepared as described above by 2-fold serial dilutions in fresh growth medium, and 50 μl was transferred into the wells of a 96-well plate in duplicate. Confluent E. histolytica trophozoites were diluted in growth medium to a density of 200 cells/μl. Fifty-microliter cultures were distributed in each well to bring the total assay volume to 100 μl. The plates were kept under an anaerobic environment for 72 h at 37°C. Each well was surveyed under a microscope to visualize trophozoite survival, mobility, and attachment. In all cases, duplicate measurements yielded identical growth/death transitions. The plates were chilled on ice for 30 min, and the entire content of each of 4 wells within the growth/death transition of one of the duplicate experiments was transferred for proliferation into 8-ml tubes containing growth medium and no drug. The tubes were incubated under anaerobic condition for 3 days at 37°C and checked under a microscope. The MLC value was the concentration in the tube with the lowest compound concentration without any live organism.

### CHO cell cytotoxicity: IC_50_ determination.

CHO cells (180 μl) were seeded at a density of 5,000 cells/well and incubated for 24 h at 37°C in a 5% CO_2_ incubator in a 96-well plate. The cells were treated with 20 μl fumagillin or fumagillol derivative solutions diluted to obtain final concentrations of 100, 30, 10, 3, 1, and 0.3 μM. Puromycin was diluted from 10 through 0.03 μM. The plates were incubated for 48 h at 37°C in a 5% CO_2_ incubator. One hundred microliters per well of ATPlite luminescence reagent was added to the plates, the plates were incubated at room temperature for 30 min, and the luminescence signal was measured. The assay was performed in duplicate.

### MetAP2 fluorescence assay.

Recombinant human MetAP2 (Bio-Techne/R&D Systems) was diluted to 10 μg/ml in the assay buffer (50 mM HEPES, 0.1 mM CoCl_2_, 100 mM NaCl, pH 7.5). The substrate H-Met-Gly-Pro-7-amido-4-methylcoumarin (Bio-Techne/R&D Systems) was diluted to 500 μM with 2 μg/ml of the coupling enzyme, recombinant human dipeptidyl peptidase 4 (Bio-Techne/R&D Systems), in assay buffer. Fifty microliters of 10-μg/ml recombinant human MetAP2 was loaded into a flat-bottom black 96-well plate (Greiner Bio), and the reaction was initiated by adding 50 μl of the substrate/dipeptidyl peptidase 4 mixture. The reaction mixture was incubated at room temperature for 10 min, and the fluorescence was measured for 5 min at excitation and emission wavelengths of 380 nm and 460 nm, respectively, in the kinetic mode. For IC_50_ determination, 0.5 μl inhibitor solution was added to the reaction mixture. Serial dilutions (1:5), starting with 1 μM for fumagillin and 200 nM for compounds 3 and 9, were prepared in duplicate. The reaction rates were fitted to a 4-parameter logistic nonlinear regression model using the KaleidaGraph tool, yielding the IC_50_ values and standard error.

### Caco-2 cell permeation assay.

Passage 36 Caco-2 cells were used. The cells were cultured in Dulbecco’s modified Eagle’s medium (DMEM; 5 ml of 100 mM sodium pyruvate, 5 ml of 100× nonessential amino acids, 5 ml of penicillin-streptomycin, and 100 ml of heat-inactivated fetal bovine serum added to 385 ml of DMEM aseptically and mixed thoroughly), pH 7.4, at 37°C in 5% CO_2_. DMEM (250 μl) was added to the basal compartment of 96-well multiscreen Caco-2 cell plate consisting of high-pore-density polycarbonate (Millipore) with a 0.4-μm pore size and a 0.11-cm^2^ active membrane area. The cells were seeded at a density of 12,000 cells/well (0.16 × 10^6^ cells/ml) in the apical wells, with one well containing only medium as blank. The Caco-2 cell plate was placed in a CO_2_ incubator at 37°C for cell proliferation. On the day of assay, the medium was removed, washed twice with Hanks balanced salt solution (HBSS) buffer supplemented with 2% bovine serum albumin (BSA), pH 7.4, and incubated with the HBSS buffer for 30 min at 37°C. Wells with transepithelial electrical resistance values of greater than 230 Ω · cm^2^ were selected for the experiments. The test compounds and two control compounds (propranolol [high-permeation control] and atenolol [low-permeation control]) were diluted to a final concentration of 10 μM with the HBSS buffer. The assay was performed in duplicate.

### (i) Apical-to-basal permeation assay protocol.

Seventy-five microliters of the test compounds was added to the apical wells, and 250 μl of HBSS buffer with 2% BSA was added to the basal wells. The plate was incubated in a CO_2_ incubator with shaking on a plate shaker at 150 rpm for 120 min, after which 25 μl of the basal samples was collected and processed.

### (ii) Basal-to-apical permeation assay protocol.

Two hundred fifty microliters of test compounds was added to the basal wells, and 75 μl of HBSS buffer with 2% BSA was added to the apical wells. The plate was incubated in a CO_2_ incubator with shaking on a plate shaker at 150 rpm for 120 min, after which 25 μl of the apical samples was collected and processed.

For sample processing, a single-point calibration curve in HBSS buffer with 2% BSA was used. Donor samples were diluted 1:1 with HBSS containing 2% BSA, and receiver samples were diluted with 1:1 HBSS buffer and precipitated with 200 μl of acetonitrile containing internal standard. The samples were vortexed for 5 min at 1,000 rpm and centrifuged at 4,000 rpm for 10 min. One hundred microliters of the supernatant was diluted with 200 μl of water and analyzed by LC-MS/MS (with a Shimadzu ultrafast liquid chromatograph and an API 4000 LC-MS/MS system).

The apparent permeation coefficients (*P*_app_) were calculated as *P*_app_ = (*dQ*/*dT*) × (1/*C*_0_) × (1/*A*), where *dQ* is the amount permeated to the receiver compartment of the 96-well plate, *dT* is the time of compound incubation, *C*_0_ is the initial compound concentration in the donor compartment of the well, and *A* is the surface area of the monolayer.

### Analyses of compound stability.

For accelerated temperature degradation tests at 75% relative humidity and at laboratory uncontrolled relative humidity, fumagillin and compounds 3 and 9 were incubated at 37°C under either controlled or uncontrolled humidity, and the samples were analyzed by HPLC every week for 4 weeks. Measurements were made in duplicate. Area-under-the-curve values were calculated to derive the percentage of compound remaining. For pH degradation, the compounds were dissolved in DMSO and mixed with pH 3.0 buffer. The samples were incubated at 25°C for up to 48 h. Samples were analyzed by HPLC every 2 h for the first 8 h and then after 24 and 48 h.

### Mouse antigiardiasis efficacy studies.

Compound efficacy was evaluated in the mature mouse giardiasis model reported previously (18, 66). Fifteen 4-week-old C57BL/6J female mice (The Jackson Laboratory, Bar Harbor, ME) were infected with G. lamblia GS or H7 trophozoites. For each experiment, the trophozoites (500,000 trophozoites suspended in 200 μl of TYI-S-33 medium) were administered by oral gavage to 15 mice. On days 3 through 6 after infection, 10 mice were treated once daily with a specified dose of a single compound, either compound 9 or fumagillin. The remaining 5 untreated mice served as controls. The compounds were administered by oral gavage in 200 μl of TYI-S-33 medium supplemented with 0.5% DMSO. All mice were euthanized on day 7. The University of Maryland College Park IACUC approved the animal studies.

Two inches of the upper small intestine were dissected and washed with 2 ml of medium supplemented with antibiotics to produce axenized cultures (1 mg/ml each piperacillin and moxalactam and 10 μg/ml each vancomycin and polymyxin B, which is a modified mixture compared with that used earlier [18, 66]). The harvested small intestines were opened longitudinally and minced in a petri dish containing 10 ml of ice-chilled medium. The plates were placed on ice for 30 min to allow the trophozoites to detach from the intestine, and the entire content of each petri dish was transferred into a 15-ml glass tube, the volume was adjusted to 14 ml by adding the antibiotic-containing medium, and the tube was vigorously vortexed to separate the trophozoites from the intestine debris. For the following 2 h, the tubes were kept at 37°C to allow trophozoite attachment to the wall of the glass tube, after which the medium containing the intestine debris was decanted and replaced by fresh medium. The tubes were kept at 37°C for another 15 min, followed by a second medium replacement. The tubes were kept at 37°C for 6 days for proliferation. Trophozoite growth was determined once daily by monitoring the ATP content using the ATPlite reagent.

The proliferation measurements were used to calculate growth curves and the trophozoite load of drug-treated mice compared with that of untreated mice. The trophozoite load was quantified by calculating the initial population from the growth curves and fitting the luminescence or the trophozoite counting data to the Malthusian model of exponential growth, *N_t_* = *N*_0_*e^rt^*, where *N_t_* is the signal of the population at time *t*, *r* is the growth rate, and *N*_0_ is the signal of the initial population at the end of treatment. The percentage of the load relative to the *N*_0_ for untreated mouse samples was determined. The 50% effective dose (ED_50_) was calculated by assuming a sigmoidal dose-response. The curve was fit with a variable slope using GraphPad Prism (version 8.2.1) software (GraphPad Software Inc., La Jolla, CA).

### Plasma pharmacokinetics.

Plasma intravenous (i.v.) and *per os* (p.o.) pharmacokinetic studies were performed in C57BL/6 female mice, using two groups of three mice each. Compound 9 was dissolved in 60% saline, 30% polyethylene glycol, and 10% DMSO. One group of mice received a single dose of 2 mg/kg of compound 9 intravenously via the tail vein. The second group of mice received a 6.6-mg/kg dose orally; this dose matched the therapeutic dose that cured giardiasis with no remaining proliferating trophozoites. Blood samples were withdrawn at 5, 15, and 30 min and 1, 2, 4, 8, and 24 h after administration for the i.v. treated mouse group and at the same time intervals but beginning at 15 min for the p.o. treated mouse group. The samples were extracted with 60% water-diluted acetonitrile and were frozen at −20°C until analysis.

Blank and test compound-containing samples were thawed, vortexed to ensure complete mixing of the contents, and centrifuged at 4,000 rpm at 20°C for 10 min. The supernatants were transferred into autoinjector vials. The vials were loaded, and 30-μl sample volumes were injected onto an API 4000 LC-MS/MS system. The Agilent XDB C_18_ column was used for the liquid chromatographic step at a flow rate of 0.6 ml/min. The mass spectrometer was operated in the electrospray ionization-positive mode. Curcumin served as an internal standard. The chromatograms were evaluated using Analyst (version 1.6.3) software.

### Single-dose acute toxicity determination and histopathology.

The maximum tolerated dose (MTD) determination was performed using 8- to 10-week-old C57BL/6 female mice in five groups of 6 animals each. The mice were acclimated to the study room for a week. Animals were randomized by body weight prior to being assigned to dosage groups at the outset of the study. Compound 9 was formulated in 10% DMSO and 90% (0.5%) hydroxypropyl methylcellulose (HPMC). The mice in groups 1 to 4 were administered single doses of 500-, 1,000-, 1,500-, or 2,000-mg/kg compound 9, respectively, and the fifth group received the vehicle alone. Following an up-down dosing strategy, the clinical signs and body weights of the animals in a study group were evaluated daily for 5 days before escalating or reducing the dose administered to the next group, as appropriate. The clinical observations included posture, vocalization, ease of handling, lacrimation, chromodacryorrhea, salivation, coat appearance, rearing frequency, transfer arousal, piloerection, motor movements, tail pinch, and diarrhea.

The histopathology examination included 3 mice that survived treatment with the 2,000-mg/kg dose and 3 mice that received the vehicle alone. Brain, heart, lung, spleen, and liver tissues were examined. Formalin-fixed tissues were embedded in paraffin to produce 4-μm-thick sections. The paraffin wax was removed, and the tissues were stained with Gill’s hematoxylin for 5 to 10 min. The samples were rinsed in tap water, followed by ammonia water, and stained with eosin Y for 5 min. The hematoxylin-eosin-stained tissues were dehydrated and mounted for light microscopic examination.

## Supplementary Material

Supplemental file 1
