# Supplementary material for: Discovery and Preclinical Development of Antigiardiasis Fumagillol Derivatives
Source: Antimicrob Agents Chemother. 2020 Sep 21;64(10):e00582-20. doi: 10.1128/AAC.00582-20 (PMC7508583; doi:10.1128/AAC.00582-20)
Supplement: Supplemental file 1 [file AAC.00582-20-s0001.pdf]

SUPPLEMENTAL MATERIAL

<u>Synthesis of fumagillol derivatives</u>

Compound 1: (3R,4S,5S,6R)-5-methoxy-4-((2R,3R)-2-methyl-3-(3-methylbut-2-en-1-yl)oxiran-2-yl)-1-oxaspiro[2.5]octan-6-yl (dimethylglycyl)carbamate.

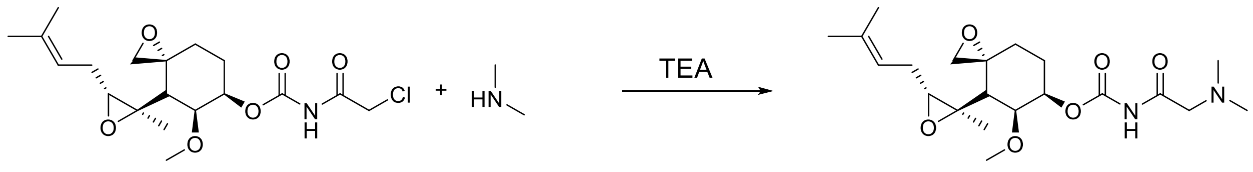

TNP-470 **1**

To a stirred solution of (3R,4S,5S,6R)-5-methoxy-4-((2R,3R)-2-methyl-3-(3-methylbut-2-en-1-yl)oxiran-2-yl)-1-oxaspiro[2.5]octan-6-yl (2-chloroacetyl)carbamate (**TNP-470**) (50 mg, 0.124 mmol) and triethylamine (12.5 mg, 0.124 mmol) in toluene (1 ml) was added dimethylamine (14 mg, 0.31 mmol) and the mixture was stirred at room temperature for 24 hrs. The solvent was removed under reduced pressure and the residue was purified by column chromatography using (eluent: 2.5% Methanol in ethyl acetate and then traces of TEA in 5% methanol in ethyl acetate) to give 10 mg of white solid (24% yield). LCMS (*m*/*z*) [M + H] 410.3 (calculated for C21H34N2O6). ^{1}H NMR (400 MHz, $CDCl_3$) δ ppm 9.21 (br s., 1 H), 5.70-5.72 (m, 1H), 5.21 (m, 1 H), 3.70 (m, 1 H), 3.45 (s, 3 H), 3.34-3.20 (m, 2H), 2.91 (s, 6H), 2.15 - 2.68 (m, 5H), 1.80-2.09 (m, 4H), 1.75 (s, 3H),1.65(s, 3H), 1.22 (s, 3H), 1.06-1.15 (m, 1H).

Compound 2: (3R,4S,5S,6R)-5-methoxy-4-((2R,3R)-2-methyl-3-(3-methylbut-2-en-1-yl)oxiran-2-yl)-1-oxaspiro[2.5]octan-6-yl (2-(piperidin-1-yl)acetyl)carbamate.

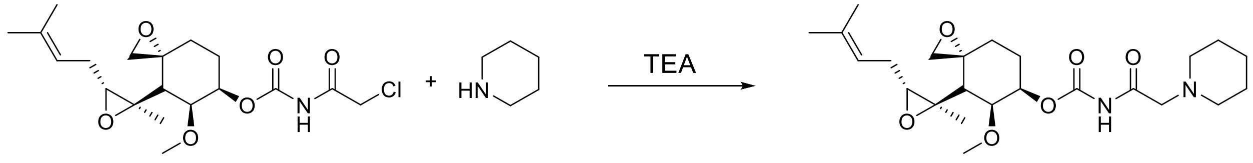

TNP-470

To a stirred solution of (3R,4S,5S,6R)-5-methoxy-4-((2R,3R)-2-methyl-3-(3-methylbut-2-en-1-yl)oxiran-2-yl)-1-oxaspiro[2.5]octan-6-yl (2-chloroacetyl)carbamate (**TNP-470**) (50 mg, 0.124 mmol) and triethylamine (12.5 mg, 0.124 mmol) in toluene (1 ml) was added piperidine (27 mg, 0.32 mmol) and the mixture was stirred at room temperature for 24 hrs. The solvent was removed under reduced pressure and the residue was purified by column chromatography using (eluent: 2.5% Methanol in ethyl acetate and then traces of TEA in 5% methanol in ethyl acetate) to give 13 mg of white solid (24% yield). LCMS (*m/z*) [M + H] 450.3 (calculated for C24H39N2O6). ^{1}H NMR (400 MHz, $CDCl_3$) δ ppm 9.32 (br s., 1 H), 5.67-5.68 (m, 1H), 5.22 (m,1 H), 3.72 (m, 1 H), 3.44 (s, 3 H), 3.30 (s, 2 H), 3.04 (m, 1 H), 2.50-2.61 (m, 4H), 2.10 - 2.68 (m, 6H), 1.95-2.09 (m, 2H), 1.75 (s, 3 H), 1.66 (s, 3H),1.45-1.60 (m, 6 H), 1.22 (s, 3 H), 1.06-1.15 (m, 1H).

Compound 3: (3R,4S,5S,6R)-5-methoxy-4-((2R,3R)-2-methyl-3-(3-methylbut-2-en-1-yl)oxiran-2-yl)-1-oxaspiro[2.5]octan-6-yl (2-morpholinoacetyl)carbamate.

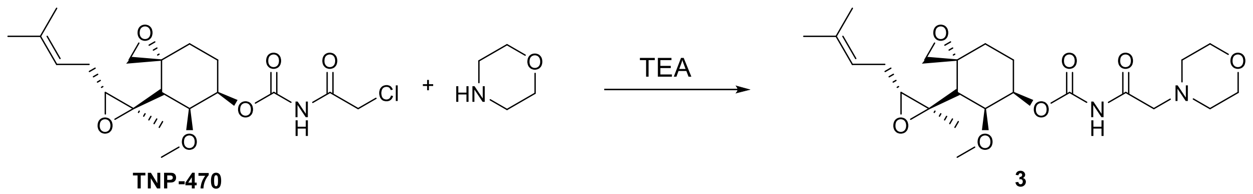

To a stirred solution of (3R,4S,5S,6R)-5-methoxy-4-((2R,3R)-2-methyl-3-(3-methylbut-2-en-1-yl)oxiran-2-yl)-1-oxaspiro[2.5]octan-6-yl (2-chloroacetyl)carbamate (**TNP-470**) (65 mg, 0.16 mmol) and triethylamine (40 mg, 0.4 mmol) in toluene (1 ml) was added morpholine (35 mg, 0.4 mmol) and the mixture was stirred at room temperature for 18 hrs. The solvent was removed under reduced pressure and the residue was purified by silica gel column chromatography (eluent: 2.5% methanol in ethyl acetate) to give 15 mg of white solid (15 mg) (21% yield).

LCMS (*m/z*) [M + H] 453.3 (calculated for C23H37N2O7). ^{1}H NMR (400 MHz, $CDCl_3$) δ ppm 5.70 (br. s., 1 H), 5.20 (t, *J*=7.46 Hz, 1 H), 3.4-3.82 (m, 7H), 3.14 (d, *J*=9.54 Hz, 2 H), 3.01 (d, *J*=4.16 Hz, 1 H), 2.55 - 2.60 (m, 5 H), 2.33-2.44 (m, 1H), 2.09-2.22 (m, 2H), 1.81-2.08 (3H), 1.75 (s, 3 H), 1.66 (s, 3 H), 1.22-1.33 (m, 3H), 1.21 (s, 3 H), 1.06-1.15 (m, 1H).

Compound 4: (3R,4S,5S,6R)-5-methoxy-4-((2R,3R)-2-methyl-3-(3-methylbut-2-en-1-yl)oxiran-2-yl)-1-oxaspiro[2.5]octan-6-yl (2-(4-hydroxypiperidin-1-yl)acetyl)carbamate.

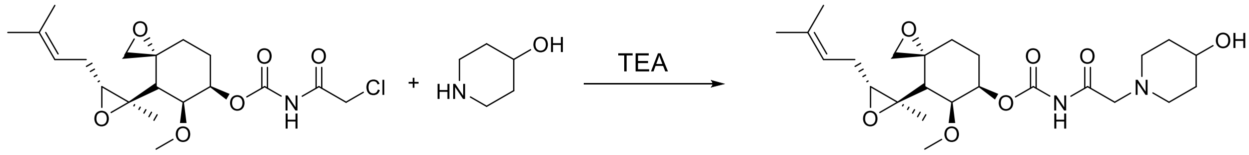

TNP-470 **4**

To a stirred solution (3R,4S,5S,6R)-5-methoxy-4-((2R,3R)-2-methyl-3-(3-methylbut-2-en-1-yl)oxiran-2-yl)-1-oxaspiro[2.5]octan-6-yl (2-chloroacetyl)carbamate (**TNP-470**) (50 mg, 0.124 mmol) and triethylamine (12.5 mg, 0.124 mmol) in toluene (1 ml) was added 4-hydroxypiperidine (41 mg, 0.31 mmol) and the mixture was stirred at room temperature for 24 hrs. The solvent was removed under reduced pressure and the residue was purified by column chromatography (eluent: 5% methanol in ethyl acetate) to give 17.4 mg of white solid (30% yield). LCMS (*m/z*) [M + H] 467.3 (calculated for C24H39N2O7). ^{1}H NMR (400 MHz, $CDCl_3$) δ ppm 9.25 (br. s., 1 H), 5.69 (br. s., 1 H), 5.21 (t, *J*=7.34 Hz, 1 H), 3.75-3.80 (m, 1H), 3.69 (dd, *J*=11.37, 2.57 Hz, 1 H), 3.48 (s, 3 H), 3.02 - 3.21 (m, 2 H), 3.01 (d, *J*=4.40 Hz, 1 H), 2.47-2.88 (m, 2 H), 2.62 (t, *J*=6.36 Hz, 1 H), 2.57 (d, *J*=4.16 Hz, 1 H), 2.27 - 2.46 (m, 3 H), 2.07 - 2.24 (m, 2 H), 1.85 - 2.03 (m, 5 H), 1.51-1.77 (m, 9H), 1.22 (s, 3H), 1.06-1.14 (m, 1H).

Compound 5: (3R,4S,5S,6R)-5-methoxy-4-((2R,3R)-2-methyl-3-(3-methylbut-2-en-1-yl)oxiran-2-yl)-1-oxaspiro[2.5]octan-6-yl (2-(4-methylpiperazin-1-yl)acetyl)carbamate.

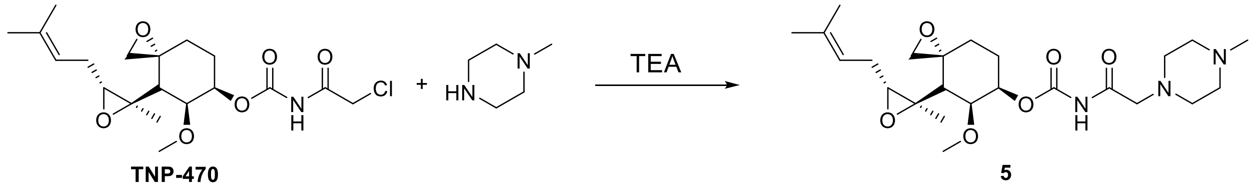

To a stirred solution (3R,4S,5S,6R)-5-methoxy-4-((2R,3R)-2-methyl-3-(3-methylbut-2-en-1-yl)oxiran-2-yl)-1-oxaspiro[2.5]octan-6-yl (2-chloroacetyl)carbamate (**TNP-470**) (50 mg, 0.124 mmol) and triethylamine (12.5 mg, 0.124 mmol) in toluene (1 ml) was added 1-methylpiperazine (31 mg, 0.31 mmol) and the mixture was stirred at room temperature for 24 hrs. The solvent was removed under reduced pressure and the residue was purified by column chromatography using (eluent: 5% Methanol in ethyl acetate and then traces of TEA in 5% methanol in ethyl acetate) to give 12.5 mg of white solid (21.6% yield). LCMS (*m/z*) [M + H] 466.3 (calculated for C24H40N3O6). ^{1}H NMR (400 MHz, $CDCl_3$) δ ppm 9.14 (br. s., 1 H), 5.70 (br. s., 1 H), 5.20 (t, *J*=7.46 Hz, 1 H), 3.69 (dd, *J*=11.25, 2.69 Hz, 1 H), 3.48 (s, 3 H), 3.34 (s, 1 H), 3.12 (d, *J*=9.54 Hz, 2 H), 3.00 (d, *J*=4.16 Hz, 1 H), 2.55 - 2.68 (m, 5 H), 2.23-2.55 (m, 8 H), 2.20-2.23 (m, 2H), 1.95-2.09 (m, 2H), 1.74 (s, 3 H), 1.54-1.69(m, 4 H), 1.22 (s, 3 H), 1.06-1.15 (m, 1H).

Compound 6: (3R,4S,5S,6R)-5-methoxy-4-((2R,3R)-2-methyl-3-(3-methylbut-2-en-1-yl)oxiran-2-yl)-1-oxaspiro[2.5]octan-6-yl (2-(4-(2-hydroxyethyl)piperazin-1-yl)acetyl)carbamate.

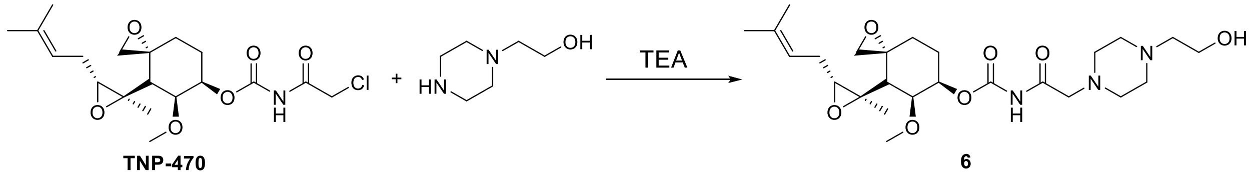

To a stirred solution (3R,4S,5S,6R)-5-methoxy-4-((2R,3R)-2-methyl-3-(3-methylbut-2-en-1-yl)oxiran-2-yl)-1-oxaspiro[2.5]octan-6-yl (2-chloroacetyl)carbamate (**TNP-470**) (50 mg, 0.124 mmol) and triethylamine (12.5 mg, 0.124 mmol) in toluene (1 ml) was added N-(2-hydroxyethyl)piperazine (40 mg, 0.31 mmol) and the mixture was stirred at room temperature for 24 hrs. S The solvent was removed under reduced pressure and the residue was purified by column chromatography using (eluent: 5% methanol in ethyl acetate) give to 37 mg of white solid (58% yield). LCMS (*m/z*) [M + H] 496.3 (calculated for C25H42N3O7).^{1}H NMR (400 MHz, $CDCl_3$) δ ppm 8.85-9.10 (br s, 1H), 5.68 (br. s., 1 H), 5.20 (t, *J*=7.46 Hz, 1 H), 3.63 - 3.73 (m, 3 H), 3.48 (s, 3 H), 3.08-3.24 (4H), 2.99 (d, J=10.6 Hz, 1H), 2.55-2.88 (m, 8H), 2.30 - 2.41(m, 1H), 2.08-2.23 (m, 2H), 1.79-2.07 (m, 3H), 1.74 (s, 3 H), 1.66 (s, 3 H), 1.42 (t, *J*=7.34 Hz, 3 H), 1.21 (s, 3 H), 1.06-1.15 (m, 1H).

Compound 7: (3R,4S,5S,6R)-5-methoxy-4-((2R,3R)-2-methyl-3-(3-methylbut-2-en-1-yl)oxiran-2-yl)-1-oxaspiro[2.5]octan-6-yl phenylcarbamate.

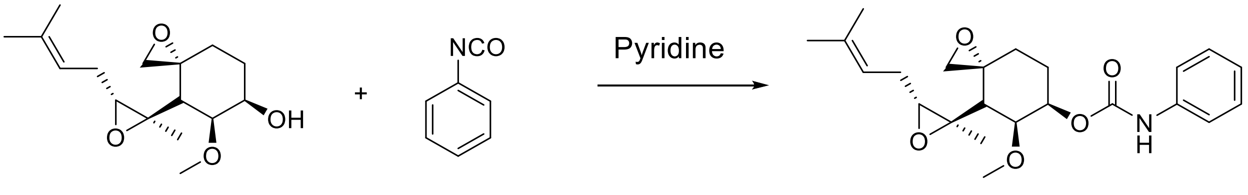

Fumagillol 7

To an ice cold stirred solution of fumagillol (56.4 mg, 0.2 mmol) in dichloromethane (5 ml) was added dropwise phenyl isocyanate (36 mg, 0.3 mmol), followed by addition of pyridine (32 mg, 0.4 mmol). The mixture was stirred at 10-15 °C for 2 hours and then allowed stir at room temperature for 15 hours. The reaction mixture was concentrated under reduced pressure. The residue was dissolved in ethyl acetate (20 ml), washed with water and saturated aqueous sodium chloride solution and dried over anhydrous magnesium sulfate. The solvent was removed under

reduced pressure and the residue was subjected to silica gel column chromatography (eluent: hexane-ethyl acetate=4:1) to give 25 mg of white solid (31 % yield).

LCMS (*m/z*) [M + H] 401.2 (calculated for C23H32NO5). ^{1}H NMR (400 MHz, $CDCl_3$) δ ppm 8.90-9.1 (m, 1H), 7.0-7.64 (m, 5H), 5.59 (br s, 1H), 5.22 (br t, 1 H), 3.71 (m, 1H), 3.42 (s, 3H), 3.00 (m, 1H), 2.5-2.69 (m, 2H), 1.62-2.33 (m, 6H), 1.75 (s, 3H), 1.66 (s, 3H), 1.23 (s, 3H), 1.07-1.17 (m, 1H).

Compound 8: Ethyl 4-(((((3R,4S,5S,6R)-5-methoxy-4-((2R,3R)-2-methyl-3-(3-methylbut-2-en-1-yl)oxiran-2-yl)-1-oxaspiro[2.5]octan-6-yl)oxy)carbonyl)amino)benzoate.

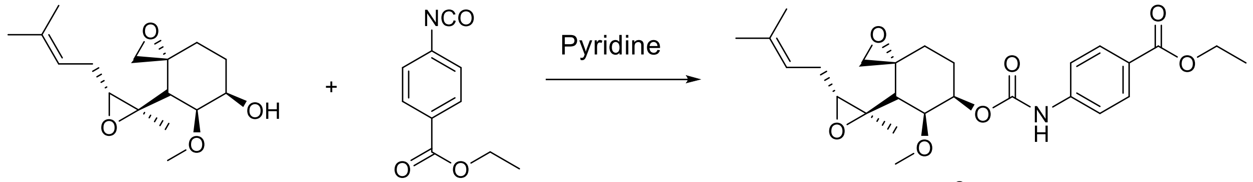

Fumagillol **8**

To an ice cold stirred solution of fumagillol (113 mg, 0.4 mmol) in dichloromethane (5 ml) was added dropwise ethyl 4-isocyanatobenzoate (115 mg, 0.6 mmol), followed by addition of pyridine (63 mg, 0.8 mmol). The mixture was stirred at 10-15 °C for 2 hours and then allowed stir at room temperature for 15 hours. The reaction mixture was concentrated under reduced pressure. The residue was dissolved in ethyl acetate (20 ml), washed with water and saturated aqueous sodium chloride solution and dried over anhydrous magnesium sulfate. The solvent was removed under reduced pressure and the residue was subjected to silica gel column chromatography (eluent: hexane-ethyl acetate=4:1) to give 121 mg of white solid (64 % yield).

LCMS (*m/z*) [M + H] 474.3 (calculated for C26H36NO7). ^{1}H NMR (400 MHz, $CDCl_3$) δ ppm 7.99-8.04 (m, 2H), 7.43-7.64 (m, 2H), 6.91 (s, 1H), 5.59 (d, 1H, j=6.3 Hz), 5.21 (br t, 1 H), 4.36 (q, 2H, j=17.8 Hz), 3.71 (dd, 1H, J=7 Hz and 28.1 Hz), 3.47 (s, 3H), 3.00 (d, 1H, J=10.7 Hz),

2.57-2.66 (m, 2H), 2.35-2.42 (m, 1H), 2.81-2.23 (m, 4H), 1.75 (s, 3H), 1.67(s, 3H), 1.56 (s, 1H), 1.38 (t, 3H, 17.8 Hz), 1.23 (s, 3H), 1.07-1.17 (m, 1H).

Compound 9: 4-(((((3R,4S,5S,6R)-5-methoxy-4-((2R,3R)-2-methyl-3-(3-methylbut-2-en-1-yl)oxiran-2-yl)-1-oxaspiro[2.5]octan-6-yl)oxy)carbonyl)amino)benzoic acid.

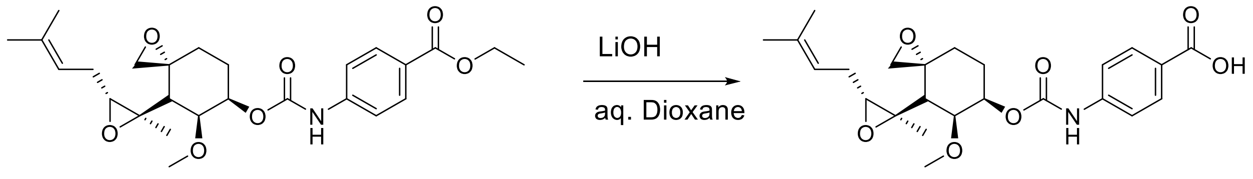

8 **9**

To a stirred solution of ethyl 4-(((((3R,4S,5S,6R)-5-methoxy-4-((2R,3R)-2-methyl-3-(3-methylbut-2-en-1-yl)oxiran-2-yl)-1-oxaspiro[2.5]octan-6-yl)oxy)carbonyl)amino)benzoate (**8**, JP-8) (95 mg, 0.2 mmol) in 2 ml of dioxane was added lithium hydroxide (48 mg, 2.0 mmol) in 0.2 ml of water. The reaction mixture was stirred for 14 hours and pH was adjusted to 5 with dilute hydrochloric acid. The reaction mixture was concentrated under reduced pressure and the residue was subjected to silica gel column chromatography (eluent: hexane-ethyl acetate=1:1 and then 100 % ethyl acetate) to give 67 mg of white solid (75 % yield).LCMS (*m/z*) [M - H] 444.2 (calculated for C24H30NO7) ^{1}H NMR (400 MHz, $CDCl_3$) δ ppm 7.90-8.00 (m, 2H), 7.36-7.52 (m, 2H), 7.03-7.21 (br s, 1H), 5.59 (br s, 1H), 5.06-5.23 (m, 1 H), 3.93-4.29 (m, 1H), 3.59-3.74 (m, 1H), 3.48 (s, 3H), 3.0-3.45 (m, 1H), 2.81-3.01 (m, 1H), 2.57-2.65 (m, 1H), 2.32-2.48 (m, 1H), 1.81-2.30 (m, 4H), 1.75 (s, 3H), 1.66(s, 3H), 1.20-1.32(m, 4H), 1.07-1.13 (m, 1H).

Compound 10: (3R,4S,5S,6R)-5-methoxy-4-((2R,3R)-2-methyl-3-(3-methylbut-2-en-1-yl)oxiran-2-yl)-1-oxaspiro[2.5]octan-6-yl (4-(4-hydroxypiperidine-1-carbonyl)phenyl)carbamate.

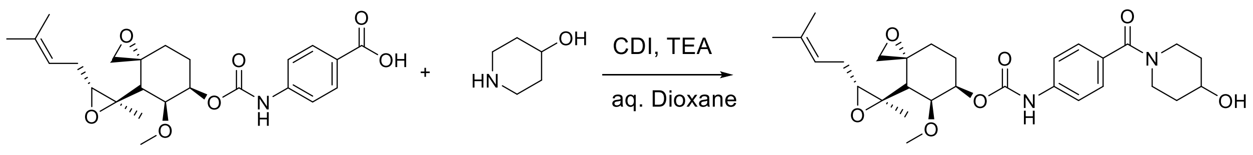

To a stirred solution of 4-(((((3R,4S,5S,6R)-5-methoxy-4-((2R,3R)-2-methyl-3-(3-methylbut-2-en-1-yl)oxiran-2-yl)-1-oxaspiro[2.5]octan-6-yl)oxy)carbonyl)amino)benzoic acid (**9,** JP-9) (49 mg, 0.11 mmol) and triethyamine (25 mg, 0.25 mmol) in DCM (5 ml) was added carbonyldiimidazole (27 mg, 0.17 mmol). The resulting reaction mixture was stirred at room temperature for 1 hour under nitrogen and then treated with a solution of piperidin-4-ol (22 mg, 0.22 mmol) in DCM (1 mL) and the stirring was continued at room temperature for 14 hours. The reaction mixture was concentrated under reduced pressure. The residue was dissolved in ethyl acetate (20 ml), washed with water and saturated aqueous sodium chloride solution and dried over anhydrous magnesium sulfate. The solvent was removed under reduced pressure and the residue was subjected to silica gel column chromatography (eluent: hexane-ethyl acetate=1:1) to give 21 mg of yellow solid (36% yield).LCMS (*m/z*) [M + H] 529.3 (calculated for C29H41N2O7) ^{1}H NMR (400 MHz, $CDCl_3$) δ ppm 8.16-8.46 (m, 2H), 7.28-7.50 (m, 2H), 5.8(d, 1H, J=5.6), 5.18 (t, 1H, J=19.3 and 17.2) 4.30 (br s, 1H), 3.79- 4.07 (m, 7H), 3.31 (s, 3H), 3.24-3.30 (m, 1H), 3.12-3.18 (m, 2H), 2.69-3.00 (m, 1H), 2.41-2.62 (m, 1H), 2.12-2.25 (m, 1H), 2.02-2.11 (m, 3H), 1.74-1.99 (m, 8H), 1.57-1.70 (m, 2H), 1.43-1.57 (m, 3H), 0.85-1.00 (m, 1H).

Compound 11: Ethyl 2-(4-(((((3R,4S,5S,6R)-5-methoxy-4-((2R,3R)-2-methyl-3-(3-methylbut-2-en-1-yl)oxiran-2-yl)-1-oxaspiro[2.5]octan-6-yl)oxy)carbonyl)amino)phenyl)acetate.

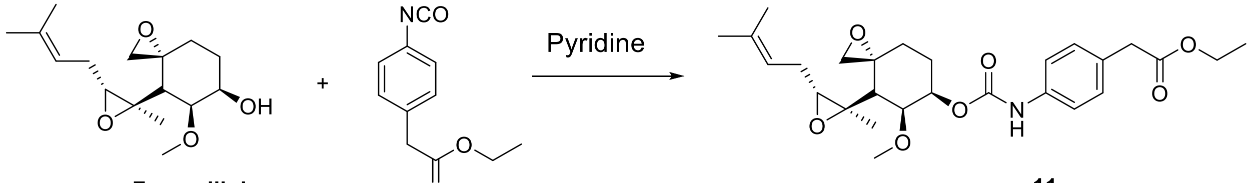

Fumagillol **11**

To a stirred ice cold solution of fumagillol (113 mg, 0.4 mmol) in dichloromethane (5 ml) was added dropwise ethyl 2-(4-isocyanatophenyl)acetate (123 mg, 0.6 mmol), followed by of pyridine (63 mg, 0.8 mmol). The mixture was stirred at 10-15 °C for 2 hours and then allowed stir at room temperature for 24 hours. The reaction mixture was concentrated under reduced pressure. The residue was dissolved in ethyl acetate (20 ml), washed with water and saturated aqueous sodium chloride solution and dried over anhydrous magnesium sulfate. The solvent was removed under reduced pressure and the residue was subjected to silica gel column chromatography (eluent: hexane-ethyl acetate=4:1) to give 111 mg of white solid (57 % yield). LCMS (*m/z*) [M+ H] 488.3 (calculated for C27H38NO7). ^{1}H NMR (400 MHz, $CDCl_3$) δ ppm 7.34 (d, 2H, j=20.8), 7.21 (d, 2H, J=20.9Hz), 6.90-7.02(br, 1H), 5.60 (br s, 1H), 5.20 (t, 1H, J=18.4Hz), 4.13 (q, 2H, j=17.8 Hz), 3.61-3.81 (m, 1H), 3.55 (s 2H), 3.44 (s, 3H), 2.99 (d, 1H, J=10.7 Hz), 2.56-2.77 (m, 2H), 2.30-2.47 (m, 1H), 1.94-2.29 (m, 4H), 1.79-1.93 (m, 1H), 1.75 (s, 3H), 1.66 (s, 3H), 1.19-1.32 (m, 6H), 1.0-1.17 (m, 1H).

Compound 12: 2-(4-(((((3R,4S,5S,6R)-5-methoxy-4-((2R,3R)-2-methyl-3-(3-methylbut-2-en-1-yl)oxiran-2-yl)-1-oxaspiro[2.5]octan-6-yl)oxy)carbonyl)amino)phenyl)acetic acid.

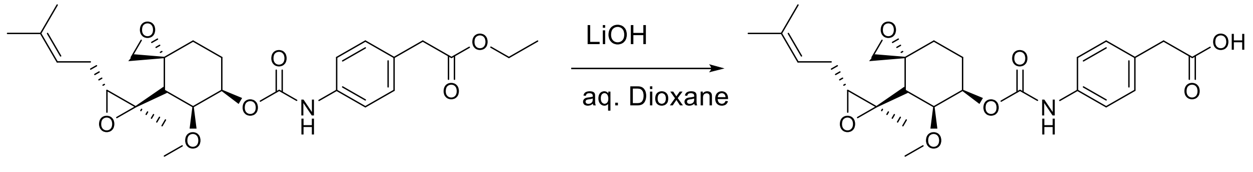

11 **12**

To a stirred solution ethyl 2-(4-(((((3R,4S,5S,6R)-5-methoxy-4-((2R,3R)-2-methyl-3-(3-methylbut-2-en-1-yl)oxiran-2-yl)-1-oxaspiro[2.5]octan-6-yl)oxy)carbonyl)amino)phenyl)acetate (**11,** JP-14) (75 mg, 0.154 mmol) in 2 ml of dioxane was added lithium hydroxide (19 mg, 0.8 mmol) in 0.2 ml of water. The reaction mixture was stirred for 24 hours and pH was adjusted to 5 with dilute HCl. The reaction mixture was concentrated under reduced pressure and the residue was subjected to silica gel column chromatography (eluent: hexane-ethyl acetate=1:1 and then 100 % ethyl acetate) to give 39 mg of white solid (55 % yield). LCMS (*m/z*) [M- H] 458.2 (calculated for C25H32NO7). ^{1}H NMR (400 MHz, $CDCl_3$) δ ppm 8.3-9.6 (br, 1H), 7.27 (d, 1H, j=21.2), 7.16 (d, 2H, j=21.2 Hz), 7.00-7.15 (br, 1H), 5.33-5.53 (m, 1H), 5.20 (t, 1H, J=18.5Hz), 3.68-3.85 (m, 1H), 3.53-3.66 (m, 2H), 3.45 (s, 3H), 2.98 (d, 1H, J=10.6 Hz), 2.60-2.77 (m, 1H), 2.56 (d, 1H, J=10.5 Hz), 2.27-2.44 (m, 1H), 1.96-2.26 (m, 4H), 1.81-1.94 (m, 1H), 1.75 (s, 3H), 1.66 (s, 3H), 1.16-1.33(m, 4H), 0.99-1.13 (m, 1H).

Compound 13: Ethyl (((3R,4S,5S,6R)-5-methoxy-4-((2R,3R)-2-methyl-3-(3-methylbut-2-en-1-yl)oxiran-2-yl)-1-oxaspiro[2.5]octan-6-yl)oxy)carbonyl)glycinate.

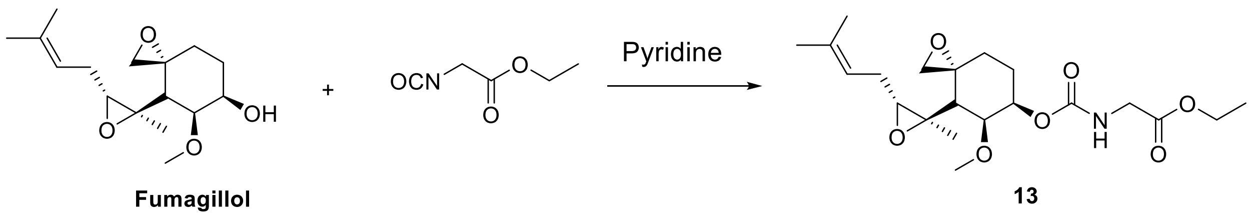

To a stirred ice cold solution of fumagillol (113 mg, 0.4 mmol) in dichloromethane (5 ml) was added dropwise ethyl 2-isocyanatoacetate (78 mg, 0.6 mmol), followed by of pyridine (63 mg, 0.8 mmol). The mixture was stirred at 10-15 °C for 2 hours and then allowed stir at room temperature for 17 hours. The reaction mixture was concentrated under reduced pressure. The residue was dissolved in ethyl acetate (20 ml), washed with water and saturated aqueous sodium

chloride solution and dried over anhydrous magnesium sulfate. The solvent was removed under reduced pressure and the residue was subjected to silica gel column chromatography (eluent: hexane-ethyl acetate=4:1) to give 94 mg of an oil (57 % yield).

LCMS (*m/z*) [M + H] 412.2 (calculated for C21H34NO7). ^{1}H NMR (400 MHz, $CDCl_3$) δ ppm 5.41-5.72 (br s, 1H), 5.29-5.36 (br t, 1H), 5.18-5.23 (br t, 1H), 4.19-4.27 (m, 2H), 3.93-4.15 (m, 1H), 3.82-3.88 (m, 1H), 3.62-3.70 (m, 1H), 3.18-3.49 (m, 3H), 2.95-3.04 (m, 1H), 2.49-2.60 (m, 2H), 2.25-2.44 (m, 1H), 1.78-2.23 (m, 5H), 1.74 (s, 3H), 1.66 (s, 3H), 1.24-1.34 (m, 3H), 1.13-1.24 (m, 3H), 0.97-1.12 (m, 1H).

Compound 14: ((((3R,4S,5S,6R)-5-methoxy-4-((2R,3R)-2-methyl-3-(3-methylbut-2-en-1-yl)oxiran-2-yl)-1-oxaspiro[2.5]octan-6-yl)oxy)carbonyl)glycine.

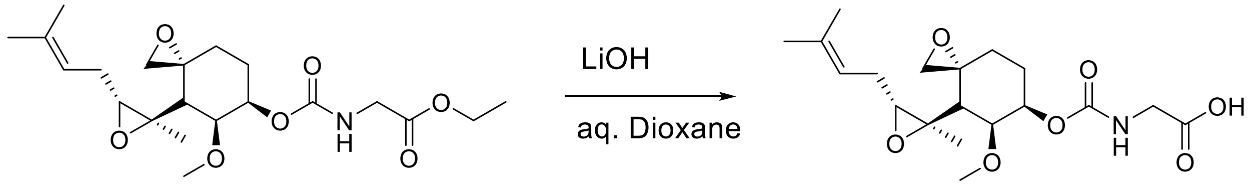

13 14

To a stirred solution of ethyl ((((3R,4S,5S,6R)-5-methoxy-4-((2R,3R)-2-methyl-3-(3-methylbut-2-en-1-yl)oxiran-2-yl)-1-oxaspiro[2.5]octan-6-yl)oxy)carbonyl)glycinate (**13,** JP-12) (62 mg, 0.15 mmol) in 2 ml of dioxane was added lithium hydroxide (18 mg, 0.75 mmol) in 0.2 ml of water. The reaction mixture was stirred for 16 hours and pH was adjusted to 5 with dilute HCl. The reaction mixture was concentrated under reduced pressure and the residue was subjected to silica gel column chromatography (eluent: hexane-ethyl acetate=1:1 and then 100 % ethyl acetate) to give 34 mg of sticky solid (59 % yield).LCMS (*m/z*) [M- H] 382.2 (calculated for C19H28NO7), ^{1}H NMR (400 MHz, $CDCl_3$) δ ppm 5.60-5.71 (br s, 1H), 5.30-5.34 (br t, 1H), 5.17-5.22 (br t, 1H), 3.99-4.11 (m, 2H), 3.59-3.84 (m, 1H), 3.40-3.52 (m, 3H), 2.90-3.05 (m,

1H), 2.47-2.63 (m, 2H), 2.30-2.44 (m, 1H), 1.6-2.31 (m, 6H), 1.76 (s, 3H), 1.64 (s, 3H), 1.23-1.33 (m, 3H), 1.01-1.1 (m, 1H).

Intermediate: (3R,4S,5S,6R)-5-methoxy-4-((2R,3R)-2-methyl-3-(3-methylbut-2-en-1-yl)oxiran-2-yl)-1-oxaspiro[2.5]octan-6-yl (4-nitrophenyl) carbonate.

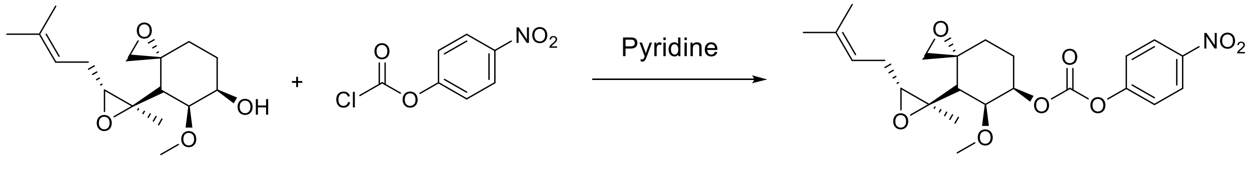

To an ice cold stirred solution of fumagillol (141 mg, 0.5 mmol) and pyridine (59 mg, 0.75 mmol) in dichloromethane (5 ml) was added slowly 4-nitrophenyl chloroformate (121 mg, 0.6 mmol). The mixture was stirred at 10-15 °C for 1 hour and then 4 hours at room temperature for 4 hours. The reaction mixture was diluted with dichloromethane (10 ml), washed with water and saturated aqueous sodium chloride solution and dried over anhydrous magnesium sulfate. The solvent was removed under reduced pressure and the residue was subjected to silica gel column chromatography (eluent: hexane-ethyl acetate=5:1) to give 141 mg of oil (63% yield). LCMS (*m/z*) [M + H] 448.2 (calculated for C23H30NO8). ^{1}H NMR (400 MHz, $CDCl_3$) δ ppm: 8.32-8.20 (d, 2H), 7.42-7.40 (d, 2H), 5.49-5.53 (m, 1H), 5.20-5.30 (m, 1H), 3.92-3.98 (m, 1H), 3.51 (s, 3H), 3.19-3.23 (m, 1H), 2.55-2.60 (m, 2H), 2.3-2.35 (m, 1H), 1.97-2.16 (m, 5H), 1.74 (s, 3H), 1.67 (s, 3H), 1.31 (s, 3H), 0.85-0.92 (m, 1H).

Compound 15: (3R,4S,5S,6R)-5-methoxy-4-((2R,3R)-2-methyl-3-(3-methylbut-2-en-1-yl)oxiran-2-yl)-1-oxaspiro[2.5]octan-6-yl piperidine-1-carboxylate.

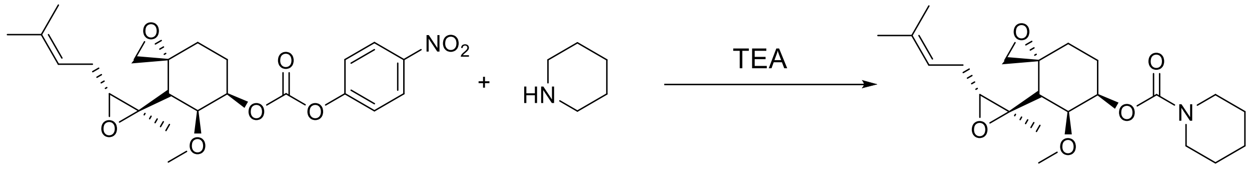

Intermediate **15**

To a stirred reaction mixture of (3R,4S,5S,6R)-5-methoxy-4-((2R,3R)-2-methyl-3-(3-methylbut-2-en-1-yl)oxiran-2-yl)-1-oxaspiro[2.5]octan-6-yl (4-nitrophenyl) carbonate (**Intermediate**) (45 mg, 0.1 mmol) and trimethylamine (40 mg, 0.4 mmol) in dichloromethane (3 ml) was added piperidine (34 mg. 0.4 mmol). The mixture was stirred for 15 hours at room temperature. The reaction mixture was concentrated under reduced pressure. The residue was dissolved in ethyl acetate (15 ml), washed with water and saturated aqueous sodium chloride solution and dried over anhydrous magnesium sulfate. The solvent was removed under reduced pressure and the residue was subjected to silica gel column chromatography (eluent: hexane-ethyl acetate=1:1) to give 14 mg of oil (36% yield).LCMS (*m/z*) [M+ H] 393.5 (calculated for C22H36NO5). ^{1}H NMR (400 MHz, $CDCl_3$) δ ppm 5.55-5.65 (br s, 1H), 5.25 (s, 1H), 3.54-3.70 (m, 1H), 3.44 (s, 3H), 2.92-3.45 (m, 5H), 2.55-2.61 (m, 1H), 1.76-2.45 (m, 7H), 1.74 (s, 3H), 1.67 (s, 3H), 1.46-1.59 (m, 6H), 1.21 (s, 3H), 1.02-1.17 (m, 1H).

Compound 16: (3R,4S,5S,6R)-5-methoxy-4-((2R,3R)-2-methyl-3-(3-methylbut-2-en-1-yl)oxiran-2-yl)-1-oxaspiro[2.5]octan-6-yl 4-hydroxypiperidine-1-carboxylate.

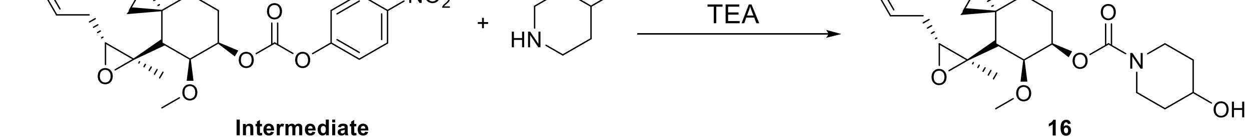

Intermediate **16**

To a stirred reaction mixture of (3R,4S,5S,6R)-5-methoxy-4-((2R,3R)-2-methyl-3-(3-methylbut-2-en-1-yl)oxiran-2-yl)-1-oxaspiro[2.5]octan-6-yl (4-nitrophenyl) carbonate (**Intermediate,** JP-6)(90 mg, 0.2 mmol) and trimethylamine (81 mg, 0.8 mmol) in dichloromethane (5 ml) was added piperidin-4-ol (81mg. 0.8 mmol). The mixture was stirred for 15 hours at room temperature. The reaction mixture was concentrated under reduced pressure. The residue was dissolved in ethyl acetate (20 ml), washed with water and saturated aqueous sodium chloride solution and dried over anhydrous magnesium sulfate. The solvent was removed under reduced pressure and the residue was subjected to silica gel column chromatography (eluent: hexane-ethyl acetate=1:1) to give 26 mg of oil (32% yield).LCMS (*m/z*) [M+ H] 410.2 (calculated for C22H36NO6). ^{1}H NMR (400 MHz, $CDCl_3$) δ ppm 5.50 (br s, 1H), 5.30(s, 1H), 4.97-5.23 (br t, 1H), 3.71-4.19, (br m, 3H), 3.54-3.70 (m, 1H), 3.45 (s, 3H), 3.04-3.32 (m, 2H), 2.99 (d, 1H, J=10.8 Hz), 2.51-2.68 (m, 2H), 2.27-2.45 (m, 1H), 2.10-2.26 (m, 1H), 1.79-2.08 (m, 6H), 1.74 (s, 3H), 1.66 (s, 3H), 1.36-1.59 (m, 2H), 1.21 (s, 3H), 1.02-1.17 (m, 1H).

Compound 17: (3R,4S,5S,6R)-5-methoxy-4-((2R,3R)-2-methyl-3-(3-methylbut-2-en-1-yl)oxiran-2-yl)-1-oxaspiro[2.5]octan-6-yl 4-phenylpiperazine-1-carboxylate.

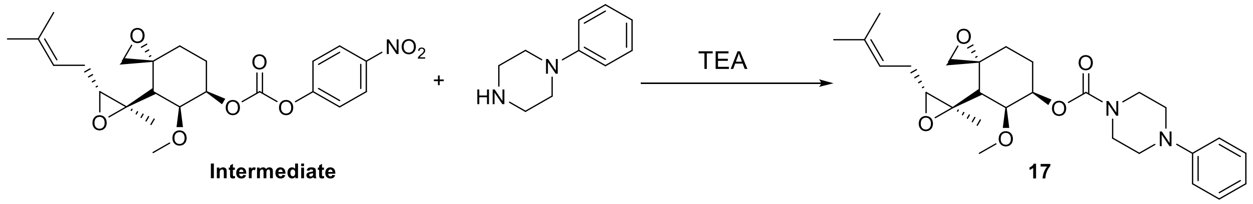

To a stirred reaction mixture of (3R,4S,5S,6R)-5-methoxy-4-((2R,3R)-2-methyl-3-(3-methylbut-2-en-1-yl)oxiran-2-yl)-1-oxaspiro[2.5]octan-6-yl (4-nitrophenyl) carbonate (**Intermediate,** JP-6) (90 mg, 0.2 mmol) and trimethylamine (81 mg, 0.8 mmol) in dichloromethane (5 ml) was added 1-phenylpiperazine (130 mg. 0.8 mmol). The mixture was stirred for 15 hours at room

temperature. The reaction mixture was concentrated under reduced pressure. The residue was dissolved in ethyl acetate (20 ml), washed with water and saturated aqueous sodium chloride solution and dried over anhydrous magnesium sulfate. The solvent was removed under reduced pressure and the residue was subjected to silica gel column chromatography (eluent: 1 to 2.5 % methanol in ethyl acetate) to give 26 mg of oil (27% yield).LCMS (*m/z*) [M + H] 471.3 (calculated for C27H39N2O5). ^{1}H NMR (400 MHz, $CDCl_3$) δ ppm 7.24-7.34 (m, 2H), 6.8-7.00 (m, 3H), 5.15-5.60 (m, 3H), 3.84-4.01 (m, 1H), 3.61-3.3.8 (br s, 4H), 3.3-3.5 (m, 3H), 3.10-3.27 (br s, 4H), 1.84-2.7, 9H), (1.60-180, m, 6H), 1.2-1.32 (m, 2H), 0.93-1.03 (m, 1H).

Compound 18: Ethyl 2-(((3R,4S,5S,6R)-5-methoxy-4-((2R,3R)-2-methyl-3-(3-methylbut-2-en-1-yl)oxiran-2-yl)-1-oxaspiro[2.5]octan-6-yl)oxy)acetate.

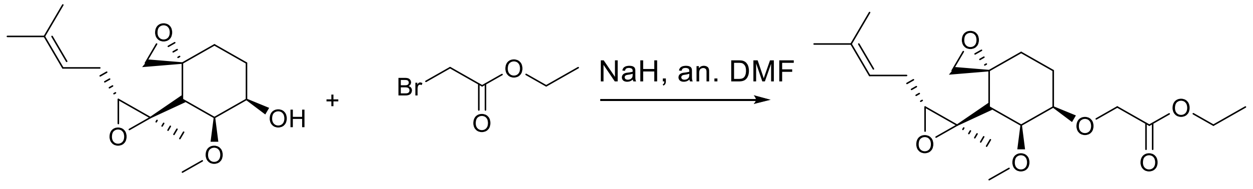

Fumagillol **18**

To a stirred ice cold solution of fumagillol (226 mg, 0.8 mmol) in anhydrous DMF (5 ml) was added 60% sodium hydride (46 mg, 1.2 mmol) followed by a slow addition of ethyl 2-bromoacetate (200 mg, 1.2 mmol) in 0.5 ml of anhydrous DMF. The mixture was stirred at 10-15 °C for 1 hours and then allowed stir at room temperature for 16 hours. The reaction mixture was concentrated under reduced pressure. The residue was dissolved in ethyl acetate (40 ml), washed with water and saturated aqueous sodium chloride solution and dried over anhydrous magnesium sulfate. The solvent was removed under reduced pressure and the residue was subjected to silica gel column chromatography (eluent: hexane-ethyl acetate=4:1) to give 146 mg of oil (49 % yield). LCMS (*m/z*) [M + H] 368.2 (calculated for C20H33O6). ^{1}H NMR (400 MHz, $CDCl_3$) δ

ppm 5.12-5.51 (br t, 1H), 4.08-4.39 (m, 4H), 3.57-3.65 (m, 1H), 3.48 (s, 3H), 2.78-3.01 (m, 1H), 2.46-2.78 (m, 2H), 2.31-2.44 (m, 1H), 2.03-2.28 (m, 3H), 1.74 (s, 3H), 1.65 (s, 3H), 1.23-1.34 (m, 3H), 1.23-1.34 (t, 3H, j=17.8), 1.20 (s, 3H), 0.96-1.05 (m, 1H).

Compound 19: 2-(((3R,4S,5S,6R)-5-methoxy-4-((2R,3R)-2-methyl-3-(3-methylbut-2-en-1-yl)oxiran-2-yl)-1-oxaspiro[2.5]octan-6-yl)oxy)acetic acid.

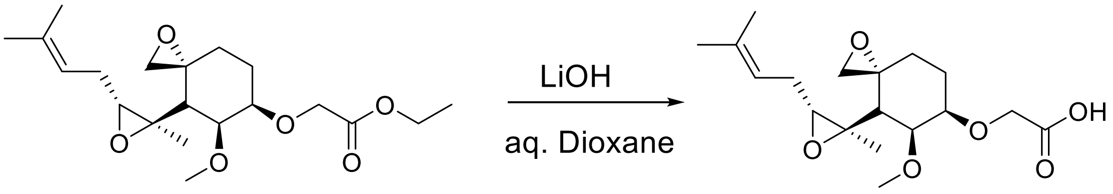

18 **19**

To a stirred solution of Ethyl 2-(((3R,4S,5S,6R)-5-methoxy-4-((2R,3R)-2-methyl-3-(3-methylbut-2-en-1-yl)oxiran-2-yl)-1-oxaspiro[2.5]octan-6-yl)oxy)acetate (**18,** JP-16) (50 mg, 0.136 mmol) in 2 ml of dioxane was added lithium hydroxide (17 mg, 0.68 mmol) in 0.2 ml of water. The reaction mixture was stirred for 24 hours and pH was adjusted to 5 with dilute HCl. The reaction mixture was concentrated under reduced pressure and the residue was subjected to silica gel column chromatography (eluent: hexane-ethyl acetate=1:1 and then 100 % ethyl acetate) to give 20 mg of white solid (44 % yield). LCMS (*m*/*z*) [M- H] 340.2 (calculated for C18H27O6). ^{1}H NMR (400 MHz, $CDCl_3$) δ ppm 10.5-11.1 (br, 1H), 5.22-5.41 (m, 1H), 4.11-4.39 (m, 2H), 3.57-3.95 (m, 5H), 2.58-3.01 (m, 3H), 1.65-2.38 (m, 6H), 1.75 (s, 3H), 1.66 (s, 3H), 1.21 (s, 3H), 0.96-1.05 (m, 1H).
